# Supplementary material for: The prevalence of non-communicable diseases in northwest Ethiopia: survey of Dabat Health and Demographic Surveillance System
Source: BMJ Open. 2017 Oct 22;7(10):e015496. doi: 10.1136/bmjopen-2016-015496 (PMC5665308; doi:10.1136/bmjopen-2016-015496)
Supplement: Author's manuscript [file bmjopen-2016-015496.draft_revisions.pdf]

BMJ Open

The burden of Chronic Non-Communicable Diseases is high in northwest Ethiopia: A population based survey at Dabat Health and Demographic Surveillance System site

Journal:	*BMJ Open*
Manuscript ID	bmjopen-2016-015496
Article Type:	Research
Date Submitted by the Author:	12-Dec-2016
Complete List of Authors:	Abebe, Solomon; University of Gondar, Institute of Public Health, College of Medicine and Health Science; University of Gondar,<br>Andarge, Gashaw ; University of Gondar, Public Health<br>Shimeka, Alemayehu ; University of Gondar<br>Alemu, Kassahun ; University of Gondar<br>Kebede, Yigzaw ; University of Gonder<br>Wubshet, Mamo; University of Gondar, Public Health<br>Woldemariam, Amare; Human Nutrition<br>Gebeyehu , Abebaw ; University of Gondar<br>Bayisa, Mulugata ; University of Gondar, School of Medicine, College of Medicine and Health Science;<br>Yitayal, Mezgebu ; University of Gondar<br>Awoke, Tadesse; University of Gondar, Epidemiology & Biostatistics<br>Azmeraw,, Temesgen ; University of Gondar<br>Birku, Melkamu ; University of Gondar
**Primary Subject Heading**:	Epidemiology
Secondary Subject Heading:	Cardiovascular medicine, Epidemiology, Public health
Keywords:	NCDs, HDSS, DM

SCHOLARONE™
Manuscripts

The burden of Chronic Non-Communicable Diseases is high in northwest Ethiopia: A population based survey at Dabat Health and Demographic Surveillance System site

Solomon Mekonnen[1], Gashaw Andargie[2], Alemayehu Shimeka[3], Kassahun Alemu[3], Yigzaw Kebede[3], Mamo Wubeshet[4,] Amare Tariku[1,] Abebaw Gebeyehu[5,] Mulugeta Bayisa[3], Mezgebu Yitayal[2], Tadesse Awoke[3], Temesgen Azmeraw[5], Melkamu Birku[5],

Authors' Information

[1]Department of Human Nutrition, Institute of Public Health, College of Medicine and Health Sciences, University of Gondar, Ethiopia
[2]Department of Health Service Management and Health Economics, Institute of Public Health College of Medicine and Health Sciences, University of Gondar, Ethiopia
[3]Department of Epidemiology and Biostatistics, Institute of Public Health College of Medicine and Health Sciences, University of Gondar, Ethiopia.
[4]Department of Environmental and Occupational Health and Safety, Institute of Public Health College of Medicine and Health Sciences, University of Gondar, Ethiopia
[5]Dabat Research Centre Health and Demographic Surviellance System, Institute of Public Health College of Medicine and Health Sciences, University of Gondar, Ethiopia
[6]Department of Physiotherapy, Institute of Public Health, College of Medicine and Health Sciences, University of Gondar, Ethiopia.
[7]Department of Reproductive Health, Institute of Public Health, College of Medicine and Health Sciences, University of Gondar, Ethiopia.

Authors Email

*SM: solomekonnen@yahoo.com
AS: alemayheushimeka@gmail.com
MB: muluphysio@yahoo.com
AT: amaretariku15@yahoo.com
GA: gashawab@gmail.com
AG: gabebaw2worku@gmail.com
YK: gkyigzaw@yahoo.com
TA:tawoke7@gmail.com
MW:mamo_wubshet@yahoo.com
TA:teme_957@yahoo.com
MB: m.berku@yahoo.com
KA: kassalemu@gmail.com

***Corresponding author**
Solomon Mekonnen
P.o.Box 196, University of Gondar

For peer review only - http://bmjopen.bmj.com/site/about/guidelines.xhtml

1. Abstract

Objective: The main objective of this study was to disclose the magnitude and associated factors thereof NCDs at a DHSS in the northwestern part of Ethiopia.

Design: A population-based cross-sectional study was conducted from October to December 2014

Setting: Dabat Health and Demographic Surveillance System (HDSS) site, Ethiopia

Participant: A total population of 67,397 living in 16,053 households was included in the study.

Measures: Structured interviewer-administered questionnaire was used to collect data. Self reported morbidity was used to ascertain NCD. A binary logistic regression model was employed to identify the determinants of NCDs.

Result: The study showed that one thousand one hundred sixty (1.7%) [95%CI: 1.62, 1.82] participants were found with at least one type of NCD. Heart disease and hypertension which accounted for 404 (32.2%) and 401 (31.9%), of the burden, respectively, were the most commonly reported NCDs, followed by 347 (27.7%) asthma, 62 (4.9%) diabetes mellitus, and 40 (3.2%) cancer cases. Advanced age (≥65 year) (AOR= 19.6; 95% CI: 5.83 65.70), urban residence (AOR= 2.20; 95% CI: 1.83, 2.65) household food insecurity (AOR= 1.71; 95% CI: 1.37, 2.12) and, high income (AOR= 1.28; 95%CI: 1.02, 1.59) were significantly associated with the reported history of NCDs, while low (AOR= 0.36, 95% CI: 0.31, 0.42) and moderate (AOR=0.33 95% CI: 0.22, 0.48) alcohol consumption, , farming occupation (AOR= 0.72; 95% CI: 0.57, 0.91), and work related physical activities (AOR= 0.66; 95% CI: 0.50, 0.88) were inversely associated with NCDs.

Conclusions: There is a high burden of NCDs at the Dabat HDS site. Therefore, promotion of regular physical exercise and reducing alcohol consumption are essential to mitigate the burden of NCDs. In addition, preventive interventions for NCDs should be strengthened for urban dwellers, aged and better-off people.

STRENGTHS AND LIMITATIONS OF THE STUDY

- This is the first community-based Prevalence study examining the magnitude and associated factors thereof of NCDs at a health and demographic site in Ethiopia.
- The stringent inclusion of all age groups
- Our study assessed the prevalence of NCDs and factors associated with it.
- Our findings provide important information that can be used to improve screening and care through medical evaluation to detect and manage NCDs.
- The limitation of this study is the use of self-report of NCDs, that may introduce recall and social desirability biases, and the morbidities reported might not be accurate diagnosis of disease conditions.
- Another limitation of this study is that it used only a cross-sectional design which might not show temporal relationships, thus, the observed associations might not necessarily be causal.

For peer review only - http://bmjopen.bmj.com/site/about/guidelines.xhtml

Introduction

2. Background

The burden of non-communicable chronic diseases (NCDs) is rising rapidly and has now become a major challenge to global development[1]. Globally, it is the leading cause of death, killing more people than all other causes each year. Disproportionately, low and middle income countries are also suffering from the consequences of these diseases [2].

There are significant social, economic, and health losses as a result of the increasing prevalence of NCDs. Even though crisis due to NCDs can be deterred through cost-effective and feasible interventions, around 80% of the deaths in these countries are caused by these diseases [3].

Non-communicable diseases have not received equal attention with communicable diseases in middle and low-income countries [4]. There is inadequate information with regard to the epidemiology of NCDs in developing countries for governments to develop NCD related action plans leaving major policy gaps for intervention. Hence country specific epidemiological and experimental studies are required for a better understanding of the burden and causes of NCDs, particularly on diabetes mellitus (DM), cardio vascular diseases (CVD), hypertension(HTN), cancers and chronic respiratory diseases (CRD) [2].

In Ethiopia, where health outcomes are highly dependent on cultural values, strong research is needed to properly assess lifestyle and anthropological measures to reveal the causes, prevention, and control of NCDs. The country is in a major economic transition, bringing new ways of life to its population. As a result, there is also an epidemiological transition in terms of diseases from infectious to non-communicable diseases attributable to modified risk factors[5]. Some of the modifiable risk factors are physical inactivity, unhealthy diet, alcohol consumption, and tobacco use, while the availability of these products is increasing. To halt the increasing prevalence of these diseases, a comprehensive multidisciplinary research is vital to elucidate the root causes and trends in the epidemiological transition of increasing NCDs in Ethiopia.

Moreover, the Ethiopian health care system is not designed for chronic health care, and health care costs related to NCDs are significant. NCD contribute to the nation's burden of poverty, slows down development, and can increase the health inequities within the country, which is believed to impose a huge demand on the healthcare system, creating economic pressure in the

For peer review only - http://bmjopen.bmj.com/site/about/guidelines.xhtml

country. In Ethiopia, NCD prevention and control remains inadequate despite the growing burden of the disease. The few studies conducted in Ethiopia are inconclusive in revealing the epidemiology of the diseases [6]. Besides, almost all of the studies done are institution-based and do not expose the prevalence at the population level. This has negatively contributed to policy gaps leading to government investing less on prevention and treatment of NCDs [7].

To understand the true burden of NCDs in Ethiopia, population based surveys are essential. Besides, it is time to assess the magnitude of NCDs and its associated factors at the population level for easy and cost-effective prevention.

3. Objective: The main objective of this study was to disclose the magnitude and associated factors of NCDs at a DHSS in the northwestern part of the country.

Methods

4. **Study Design**

A population based cross sectional study was conducted from October to December 2014 at the Dabat HDSS site, Dabat District, northwest Ethiopia.

5. **Setting:** The district is located 820 km from Addis Ababa, the capital of Ethiopia. Based on the 2007 national census conducted by the Central Statistical Agency (CSA) [8], the district has a total projected population of 216,240 with an area of 1,187.93 square km. Dabat has a population density of 122.49, which is greater than the zonal average of 63.76 persons per square km.

The Dabat Health and Demographic Surveillance System was established in 1995 in the rural district of Dabat, hosted by the University of Gondar. In Ethiopia, there is inadequate vital event registration system to date; instead, population and housing census, national sample surveys, and health facility reports are the main sources, of information on population health. Thus, Dabat HDS was established in order to produce a longitudinal data to aid in better health policy formulation, evidence based decision making, and health practice that the University of Gondar has established.

For peer review only - http://bmjopen.bmj.com/site/about/guidelines.xhtml

6. **Participants:** At the moment, 67,397 inhabitants live in 16,053 households in a randomly selected 13 (9 rural and 4 urban) kebeles (the lowest administrative units) of the HDSS site. Household level data on the general socio-demography of family members, housing and environmental conditions, food security, chronic diseases, risk factors for non-communicable diseases, injury in the last two weeks, maternal and child health, are updated every six months that is, twice a year. All permanent residents in the DHS area were eligible to participate in the study. The majority of the residents live on subsistence agriculture, producing mainly food crops.
7. **Data sources/ measurement:** Structured, interviewer administered questionnaire was used to collect data from the HDS survey site on reported history of blood pressure, diabetes, cancer, heart disease, asthma, and history of other risk factors for chronic disease conditions (alcohol consumption, cigarette smoking, vegetable consumption, and physical activity). Data was collected by diploma gradute professionals who were experienced in data collection at the HDS center. The questionnaire was developed by considering questions used previously at this site. The English version of the questionnaire was translated to Amharic and back translated to English by language experts for maintaining the consistency of meaning.
8. **Sample size and sampling technique:** All permanent residents (67,397) of the HDSS site living in 16,053 households were included in the study. This study has used all the permanent residents at Dabat HDS research center. The heads of households, mostly mothers were interviewed to capture data on events (NCDs) that happened in the families. Variables: The outcome of this study, NCD, was ascertained using the reported history of having at least one of the NCDs, such as diabetes mellitus, hypertension, cancer, asthma and heart disease prior to data collection. Accordingly, a household member was labeled as having non-common chronic diseases if they had at least one reported NCD type. Socio-demographic characteristics (age, sex, ethnicity, religion, marital status, residence, wealth index and family size), food security status, and other health related variables were considered as independent variable.
9. **Quantitative variables:** Food security is defined as existing when "all people, at all times, have physical and economic access to sufficient, safe, and nutritious food to meet their dietary needs and food preferences for an active and healthy life". In order to assess

For peer review only - http://bmjopen.bmj.com/site/about/guidelines.xhtml

the food security status of the households, an 18 item community food insecurity access scale assessment tool was adopted from Household Food Insecurity Access Scale (HFIAS) Indicator Guide VERSION 3 and categorized in four levels using HFIS variables. A frequency of occurrence question was asked to determine whether the condition happened rarely (once or twice), sometimes (three to ten times), or often (more than ten times) in the past four weeks, to find out whether participants were food secure, mildly food insecure, moderately food insecure, severely food insecure.

The household wealth index was computed for urban and rural residents separately, using the Principal Component Analysis (PCA). Urban wealth status was calculated by considering properties like selected household assets while only tropical livestock unit was used for rural residents. In the PCA, an Eigen value of greater than one, KMO distribution, and in the final model, common factor scores were summed and ranked into lowest, middle, second, fourth, and highest.

10. **Data quality control measures:** The quality of data was insured by pre-testing the data collection tools on 5% of the participants in Debark district which has a similar socio-demographic characteristic with the study district. Data was collected by 26 diploma grduate professionals and 5 individuals supervised the process. Supervision was also carried out at the spot by the research center leaders. Data collectors and supervisors had a long time experience working for the Dabat DHS site. Training was given to both data collectors and supervisors on questionnaire, content interviewing technique, purpose of the study and maintain confidentiality of information. The collected data were checked for completeness and clarity by the assigned supervisors and the principal investigator.

11. **Data Analysis Procedures:** Data were entered in to the HRS 2 software and exported to STATA version 14 for further analysis. The overall prevalence of NCD was defined as the presence of any of the studied non-communicable chronic diseases on interview among the study population. The results were presented in the form of tables, figures, and summary statistics. Logistic regressions were used to assess the association of various factors and the outcome variable. The Bi-variable analysis with a crude odds ratio of 95% CI was used to see the crude effect of independent variables on NCDs. All independent variables that had a p-value of 0.20 in the Bi-variable analysis were fitted to the multivariable logistic regression analysis to control possible confounding effects of

For peer review only - http://bmjopen.bmj.com/site/about/guidelines.xhtml

variables. Variables which were found to be significant at a p-value 0.05 level and 95% CI were considered to be the determinant factors of NCDs.

12. **Ethical considerations**: Ethical clearance was obtained from the Ethical Review Board (IRB) of the University of Gondar before starting the actual data collection. Then, participants in the selected kebeles were informed about the purpose of the study, the importance of participation, and their right to withdraw at any time. Informed written consent was obtained prior to data collection. Privacy and confidentiality of information given by each respondent was kept properly, names were not recorded, and the recorded data were kept in a secured place with strict confidentiality. Individuals identified as cases of NCDs were referred to the nearby clinic for further treatment and follow-up.

Results

13. **Participants:** A total of 67,397 people were enrolled in this study. Women comprised about 50.77%. The majority (75.10%) of the study population lived in areas. Among the study subjects, 42.96% (28,952) were under 15 years of age. Christianity is the dominant religion, accounting for 96.36%of the participants. The socio-demographic characteristics of the study population are depicted in Table 1.

14. **Descriptive data:** About 8.87% of the participants never drank alcohol in their life; while 88.2% and 2.88% were occasional and frequent consumers, respectively. About 99.5% of the study participants never smoked cigarette in their lifetime and 44.7% performed vigorous work related to physical activity on daily bases (Fig1 and 2).

    Figure 1: Reported alcohol intake, smoking and physical activity by age group of Dabat HDS research center population, Northwest Ethiopia, 2016

    Figure 2: Alcohol consumption of Dabat HDS research center population by alcohol intake, smoking and physical activity, Northwest Ethiopia, 2016

15. **Outcome data:** Participants who had NCDs were 1160 (1.7%) [95%CI: 1.62, 1.82] and according to the proportionate NCDs morbidity ratio (morbidity fraction) were 404 (32.2%) heart disease, 401 (31.9%) hypertension, 347 (27.7%) asthma, 62 (4.9%) diabetes mellitus, and 40 (3.2%) was cancer (Table 2). The mean (±SD) age of subjects with NCDs was 49.32 (±17.9). Out of the 16,035 house-holds, 1146 (6.98%) had at lest

For peer review only - http://bmjopen.bmj.com/site/about/guidelines.xhtml

For peer review only

one NCD per house hold who are residing in the HDSS and 40 (3.6%) of these had two NCDs cases per household. Of these, 746 (64.6%) were female with 48.96% in food insecure group. The prevalence of all types NCDs was slightly higher among women in all age groups.

16. **Factors:** After adjusting for a number of important covariates, the multivariable logistic regression analysis showed that increasing age, alcohol consumption, high income, living in urban areas, farming, work related physical activities, and severe food insecurity were associated with the odds of NCDs. Advanced age (≥ 65 year) (AOR= 19.6; 95% CI: 5.83 – 65.7), living in urban areas (AOR= 2.20; 95% CI: 1.83, 2.65), and severe household food insecurity (AOR= 1.71; 95% CI: 1.37, 2.12) were significantly associated with reported history of NCD while low and moderate alcohol consumption (AOR= 0.36, 95% CI: 0.31, 0.42) and (AOR=0.33 95% CI: 0.22, 0.48), high income (AOR= 1.28; 95%CI: 1.02, 1.59), farming (AOR= 0.72; 95% CI: 0.57, 0.91), and work related physical activities (AOR= 0.66; 95% CI: 0.50, 0.88) were inversely associated with NCDs (table 3).

Discussion

17. **Key result**: The number of NCD cases seen at the Dabat HDS site was largee among the elderly; the proportions were considerably high among urban dwellers. Older age, physical inactivity, living in urban areas, farming, alcohol consumption, severe food insecurity, and low income were the strongest predictors of NCDs. Another study done in rural and urban Ethiopia had issued a similar report [9]. Similar review study done in Sub-Saharan countries showed that NCDs are now the second most common causes of death after infectious diseases [10]. Our findings show that the occurrence of NCDs is significantly high among severely food insecure groups. This study is supported by a finding from Bangladesh which showed that persons with shorter stature are more likely to develop NCDs like diabetes and hypertension [11]. The WHO disease classification report shows that the disease phenotype encountered in Africa is very different from the West, and most closely resembles malnutrition-related. Some researchers also indicated that early exposure to cereal products or solid foods might increase an immune response,

For peer review only - http://bmjopen.bmj.com/site/about/guidelines.xhtml

which could trigger the destruction of β-cells[12]. Another finding shows people who had been small at birth and grown least during the first year of life had a chance of developing NCDs in the later life [13]. This disease burden can affect the normal growth of the people there-by developing early onset of non-communicable diseases in early-life and childhood. This exposure is likely to be responsible for the observed high rate of prevalence in this low economic status group [14].

Likewise, in our study a high level of NCDs were noted among individuals with high income. The findings is consistent with other [12]. The likely explanations suggested that people with butter economic statuswill be affected by mechanization and un-healthy eating lifestyle that keep people indoors, which could expose them to low physical activity that will lead to excess prevalence of NCDs [12, 15]. These data suggest that the burden of NCDs is increasing perhaps as a result of demographic and epidemiological changes. Even though the country is undergoing a delayed transition, the burden of both malnutrition related and non-communicable diseases remain high [16, 17]. This double burden has a negative implication on major economic transitions [18].

The effect of ageing on NCDs prevalence is already evident in Africa [19]. Aging causes a progressive decline in strength of musculature, which causes muscle atrophy, the risk of developing CVD. In our finding the prevalence of NCDs increases with increasing age. This finding is consistent with other reports from India, [12] as NCD epidemic matures, the age at onset will shift to younger age-groups. At aging many more people are exposed to the risk factors for long periods for the complications to develop and for them to experience the clinical syndromes of NCDs [16]. Unless action is taken for elderly people in Ethiopia, delayed due to lack of awareness, accessibility, distance to facilities, and the affordability of the health services for elderly population will delayed diagnosis [20].

Physical activity is more common in rural than urban regions of Africa because the rural population often rely on intense agricultural activities as their main occupation. In line with this, our finding shows that the prevalence of NCDs is low among farmers and physically active individuals which is consistent with the met-analysis done in China,

For peer review only - http://bmjopen.bmj.com/site/about/guidelines.xhtml

Ghana, Mexico, India, Russia, and South Africa about the risk factors of chronic non-communicable diseases [21]. Sedentary life style and lack of physical activities accelerate the occurrence of NCDs; this condition is a growing challenge to sub-Saharan countries [22, 23]. Walking time and space are drastically reduced in an urban community as compared with a rural community. This reduction in physical activity associated with life in a city, partly explains the excessive prevalence of NCDs in urban areas. studies show that lack of physical activity appears to be a significant risk factor for CVD in Sub-Saharan Africa [12, 16]. Reductions in physical activity and more sedentary modes of living parallel nutrition transition, producing an overall positive energy balance, thereby a paradigm of 'lifestyle transition [24].

In developing countries, rapid and unplanned urbanization and modernization resulting in socio-economic and behavioral changes in people directly affects the health of the urban population [16, 25]. The main difference in physical activity between the two types of community, however, is the use of walking in rural areas as a means of transportation. A diet high in total fat, cholesterol, sugar and other refined carbohydrates, and low in polyunsaturated fatty acids and fiber, often accompanied by an increasingly sedentary life, characterize life in the urban community [25]. In other presumptions people exposed to periodic famines which occur in Africa, would through natural selection increase the frequency of certain genetic trait(s), "thrifty genes," which would protect against starvation during times of famine. Therefore, a continued discussion of proven cost-effective interventions for non-communicable chronic diseases, will be useful for improving public health policy [24].

18. **Limitation:** The limitation of this study is the use of self-reported NCDs, that may introduce recall and social desirability biases, and the morbidities reported might not be accurate diagnosis of the disease condition, in that diseases might be miss-classified. Moreover, since this re-census was a general survey of the population, it may not provide in-depth information about specific diseases which occurred in the community. Another limitation of this study is that it used only a cross-sectional design which might not show temporal relationships, thus, the observed associations might not necessarily be causal.

For peer review only - http://bmjopen.bmj.com/site/about/guidelines.xhtml

Therefore, a longitudinal research is needed to assess the relationship between the variables over time.

19. **Conclusions:** The number of NCD cases seen at the Dabat HDS site is high among the elderly, and the proportions were considerably higher among the urban population. Effective and sustainable strategy for the prevention of chronic diseases throughout, implementing new intervention strategies by providing playground in the urban community, improving the lively-hood of the rural community and increasing access to healthy foods are highly essential.

Acknowledgement

We would like to acknowledge the University of Gondar and EPH CDC for the financial support. We are highly indebted to extend our thanks to Dabat district health offices for their permission to conduct this study. We want express our greatest thanks to all staff of the Dabat DHS and the study participants.

Authors' contributions

SM, GA, AG, YK, TA, MW, TA, MB, KA designed the study, SM, AS, MB, AT performed the analysis and interpretation of data and drafted the paper. SM, AS, MB, AT SM, GA, AG, YK, TA, MW, TA, MB, KA, revised the drafts of the paper. All authors prepared the draft manuscript, read and approved the final manuscript.

Funding: There are no funders to report for this submission

The authors declare no competing interests.

Data sharing statement: No additional data are available.

For peer review only - http://bmjopen.bmj.com/site/about/guidelines.xhtml

References

1. Habib SH, S.S., *Burden of non-communicable disease: Global overview. .* Diabetes Metab Syndr Clin Res Rev. , 2010. **4** (1): p. 41-47.
2. Miranda, J.J., et al., *Non-communicable diseases in low- and middle-income countries: context, determinants and health policy.* Trop Med Int Health, 2008. **13**(10): p. 1225-34.
3. Kankeu, H.T., et al., *The financial burden from non-communicable diseases in low- and middle-income countries: a literature review.* Health Res Policy Syst, 2013. **11**: p. 31.
4. Boutayeb, A., *The double burden of communicable and non-communicable diseases in developing countries.* Trans R Soc Trop Med Hyg, 2006. **100**(3): p. 191-9.
5. Lee, I.M., et al., *Effect of physical inactivity on major non-communicable diseases worldwide: an analysis of burden of disease and life expectancy.* Lancet, 2012. **380**(9838): p. 219-29.
6. Mulu, A., et al., *Epidemiological and clinical correlates of malaria-helminth co-infections in Southern Ethiopia.* Malar J, 2013. **12**: p. 227.
7. Murray, C.J. and A.D. Lopez, *Global mortality, disability, and the contribution of risk factors: Global Burden of Disease Study.* Lancet, 1997. **349**(9063): p. 1436-42.
8. *Summary and statistical report of the 2007 population and housing census, Ethiopia* in *(CSA).* 2007.
9. Tesfaye, F., *Epidemiology of Cardiovascular Disease Risk Factors in Ethiopia The rural-urban gradient* in *Department of Public Health and Clinical Medicine*. 2008, Umeå University: Umeå University, Umeå 2008:2004042.
10. Mbewu, A. and J.C. Mbanya, *Cardiovascular Disease*, in *Disease and Mortality in Sub-Saharan Africa*, D.T. Jamison, et al., Editors. 2006: Washington (DC).
11. Hoque, M.E., M.R. Khokan, and W. Bari, *"Impact of stature on non-communicable diseases: evidence based on Bangladesh Demographic and Health Survey, 2011 data"*. BMC Public Health, 2014. **14**: p. 1007.
12. Yoon, J., et al., *The Non-Communicable Disease Burden in Korea: Findings from the 2012 Korean Burden of Disease Study.* J Korean Med Sci, 2016. **31 Suppl 2**: p. S158-S167.
13. Martyn, C.N. and D.J. Barker, *Reduced fetal growth increases risk of cardiovascular disease.* Health Rep, 1994. **6**(1): p. 45-53.
14. Seligman, H.K., et al., *Food insecurity and glycemic control among low-income patients with type 2 diabetes.* Diabetes Care, 2012. **35**(2): p. 233-8.

For peer review only - http://bmjopen.bmj.com/site/about/guidelines.xhtml

15. Mbanya, J.C., et al., *Diabetes in sub-Saharan Africa.* Lancet, 2010. **375**(9733): p. 2254-66.

16. Steyn, K. and A. Damasceno, *Lifestyle and Related Risk Factors for Chronic Diseases*, in *Disease and Mortality in Sub-Saharan Africa*, D.T. Jamison, et al., Editors. 2006: Washington (DC).

17. Adetunji, J. and E.R. Bos, *Levels and Trends in Mortality in Sub-Saharan Africa: An Overview*, in *Disease and Mortality in Sub-Saharan Africa*, D.T. Jamison, et al., Editors. 2006: Washington (DC).

18. Musaiger, A.O. and H.M. Al-Hazzaa, *Prevalence and risk factors associated with nutrition-related noncommunicable diseases in the Eastern Mediterranean region.* Int J Gen Med, 2012. **5**: p. 199-217.

19. Ayernor, P.K., *Diseases of ageing in Ghana.* Ghana Med J, 2012. **46**(2 Suppl): p. 18-22.

20. Sikder, S.S., et al., *Care-seeking patterns for fatal non-communicable diseases among women of reproductive age in rural northwest Bangladesh.* BMC Womens Health, 2012. **12**: p. 23.

21. Wu, F., et al., *Common risk factors for chronic non-communicable diseases among older adults in China, Ghana, Mexico, India, Russia and South Africa: the study on global AGEing and adult health (SAGE) wave 1.* BMC Public Health, 2015. **15**: p. 88.

22. Thornton, J.S., et al., *Physical Activity Prescription: A Critical Opportunity to Address a Modifiable Risk Factor for the Prevention and Management of Chronic Disease: A Position Statement by the Canadian Academy of Sport and Exercise Medicine.* Clin J Sport Med, 2016. **26**(4): p. 259-65.

23. Mbanya, J.C. and K. Ramiaya, *Diabetes Mellitus*, in *Disease and Mortality in Sub-Saharan Africa*, D.T. Jamison, et al., Editors. 2006: Washington (DC).

24. Zaman, M.M., et al., *Prevalence of risk factors for non-communicable diseases in Bangladesh: Results from STEPS survey 2010.* Indian J Public Health, 2016. **60**(1): p. 17-25.

25. C. Angkurawaranon, W.J., B. Chenthanakij, P. Doyle, D. Nitsch, *Urbanization and non-communicable disease in Southeast Asia: a review of current evidence.* Public health, October 2014. **128**(10): p. 886-895.

For peer review only - http://bmjopen.bmj.com/site/about/guidelines.xhtml

Table 1: Socio-demographic characteristics of the population of Dabat HDS research center, Northwest Ethiopia, 2016.

Variables	Frequency	Percent
Sex Male	33,181	49.23
Female	34,214	50.77
Age in years		
Under 15	28,952	42.96
15 to 24	13,289	19.72
25 to 34	8,170	12.12
35 to 44	6,322	9.38
45 to54	4,720	7.00
55 to 64	3,020	4.48
65 and above	2,918	4.33
Residence		
Rural	50,769	75.10
Urban	16,835	16.84
Marital status		
Under 10 years old	20,089	29.81
Married	21,814	32.37
Single	19,746	29.30
Divorced	2,390	3.55
Widowed	2,369	3.52
Separated	917	1.36
Cohabiting	68	0.10
Religion		
Orthodox	64,940	96.36
Muslim	2,444	3.63
Others (Catholic, Protestant)	11	0.01
Ethnicity		
Amhara	67,294	99.85
Tigre	84	0.12
Others (Oromo,Agaw)	17	0.02
Education		
Not on education (<7years)	13,672	20.29
Unable to read and write	21,149	31.38
Read and write	5,541	8.22
Grade 1-4	10,960	16.26
Grade 5-6	4,560	6.77
Grade 7-8	3,590	5.33
Grade 9-10	4,375	6.49
Grade 11-12	1,957	2.90
Grade 12 and above	1,591	2.36
Location		
Low land		
High land		

For peer review only

For peer review only - http://bmjopen.bmj.com/site/about/guidelines.xhtml

Variables	Frequency	Percent
Occupation		
No occupation(under 10 years)	20,436	38.45
Students	13,955	26.26
Farmers	12,647	23.80
Employed permanent	1,951	3.67
Private job	1,167	2.20
Job seeker	1056	1.99
Merchant	656	1.23
House maid	623	1.17
Employed contract	328	0.62
Retired	296	0.56
Others(housewife, shepherd, disabled)	33	0.05
Location		
Low land	22,380	66.9
High land	45,224	33.1
Relation to the HH head		
HH head	16,082	23.86
Housewife	10,542	15.64
Son/daughter	34,702	51.49
Sister/brother	538	0.80
Mother/father	310	0.46
Grandson/granddaughter	3095	4.59
Other relative	1196	1.77
Other non-relative	930	1.38
Family size		
1-4	24,512	36.35
5-9	41,667	61.79
10-15	1,250	1.85
Wealth status		
Poorest quintile	9,475	14.58
Second quintile	11,344	17.45
Third quintile	13,031	20.05
Fourth quintile	14,593	22.45
Richest Quintile	16,577	25.47

For peer review only

For peer review only - http://bmjopen.bmj.com/site/about/guidelines.xhtml

Table 2: Proportion of Chronic Non-Communicable Diseases by age, and sex among Dabat HDS sit population, Northwest Ethiopia, 2016.

Variable	Gender	Reported cases age in Year n(%)						
		1-14	15-24	25-34	35-44	45-54	55-64	≥ 65
Diabetes mellitus	Female	1(0.01)	5 (0.08)	2 (0.05)	3(0.09)	3 (0.12)	7 (0.45)	5 (0.38)
	Male	4 (0.03)	7 (0.10)	7 (0.18)	5 (0.17)	4 (0.19)	4 (0.28)	5 (0.31)
Hypertension	Female	1 (0.010)	6(0.09)	22 (0.50)	30 (0.91)	49(1.90)	67 (4.26)	86 (6.56)
	Male	2 (0.01)	4 (0.06)	3 (0.08)	15 (0.50)	24 (1.12)	29 (2.0)	60 (3.73)
Heart Disease	Female	10 (0.07)	31(0.47)	54 (1.24)	50 (1.52)	66 (2.56)	43 (2.74)	47 (3.59)
	Male	4 (0.030)	12 (0.18)	14 (0.37)	14 (0.46)	19 (0.89)	12 (0.83)	27 (1.68)
Asthma	Female	2(0.01)	7(0.11)	32 (0.73)	41(1.24)	44(1.71)	36(2.29)	31(2.37)
	Male	4(0.03)	6(0.09)	21(0.55)	33(1.09)	32(1.50)	29 (2.0)	28(1.74)
Mental health	Female	12 (0.08)	26 (0.39)	27 (0.62)	18 (0.55)	19 (0.74)	11(0.70)	9(0.69)
	Male	11 (0.08)	21 (0.31)	16 (0.42)	14 (0.46)	16 (0.75)	3 (0.21)	5 (0.31)
Cancer	Female	2 (0.01)	0 (0.00)	2 (0.05)	6 (0.18)	10 (0.39)	0 (0.00)	6 (0.46)
	Male	1 (0.01)	2(0.03)	1(0.03)	1(0.03)	1 (0.05)	5 (0.35)	3 (0.19)

For peer review only - http://bmjopen.bmj.com/site/about/guidelines.xhtml

Table 3: Factors associated with NCDs of the population of Dabat HDS research center, Northwest Ethiopia, 2016.

Variables	NCDs case n(%)	COR [95%CI]	AOR [95%CI]
**Sex**			
Female	746 (2.18)	1.00	1.00
Male	409 (1.23)	0.83 [0.74,0.93]	0.85 [0.69 - 1.03]
**Age in year**			
0 to 14	**30 (0.10)**	**1.00**	1.00
15 to 24	78 (0.59)	2.76 [2.20,3.45]	1.55 [0.47, 5.10]
25 to34	153 (1.87)	4.17 [3.32, 5.23]	3.64[1.09- 12.13] *
35 to44	188 (2.97)	4.73 [3.73,5.99]	6.33 [1.89- 21.2]**
45 to54	233 (4.94)	7.23 [5.74,9.12]	11.2 [3.36- 37.5] **
55 to64	209 (6.92)	10.1 [7.92,12.79]	16.1[4.79 - 53.8] **
≥ 65	264 (9.05)	26.27 [21.2,32.2]	19.6 [5.83 – 65.7] **
**Alcohol consumption**			
Never	340 (1.03)	1.00	1.00
Occasional	782 (2.35)	0.50 [0.42, 0.59]	0.36 [0.31 - 0.42] **
2 to 3 times a week	33 (3.04)	0.47 [0.31, 0.71]	0.33 [0.22 - 0.48] **
**Smoking status**			
No	1,110 (2.96)	1.00	1.00
Yes	6 (3.51)	1.73 [0.85, 3.52]	0.80 [0.34 - 1.91]
**Wealth status**			
Poor income	170 (1.80)	1.00	1.00
Low income	172 (1.52)	0.67 [0.57, 0.79]	0.90 [0.71 - 1.14]
Mid-level income	175 (1.34)	0.45 [0.38,0.55]	0.91 [0.72 - 1.16]
Better income	279 (1.91)	0.48 [0.41,0.57]	1.19 [0.96 – 1.49]
High income	336 (2.03)	0.35 [0.29,0.42]	1.28 [1.02 - 1.59] *
**Residence**			
Rural	538 (1.06)	1.00	1.00
Urban	617 (3.68)	1.06 [0.93,1.20]	2.20 [1.83 - 2.65] **
**Education status**			
Illiterate	10 (0.07)	1.00	1.00
Can read &write	646 (3.05)	12.549.25,16.99]	0.85 [0.20 - 3.52]
Primary school	140 (2.53)	6.274.41,8.91]	0.87 [0.21 – 3.61]
High school	55 (0.50)	2.48 [1.72, 3.56]	1.29 [0.31 – 5.39]
Diploma and above	304 (1.89)	3.15 [2.25, 4.40]	1.43 [0.35 – 5.89]

For peer review only - http://bmjopen.bmj.com/site/about/guidelines.xhtml

VARIABLES	NCDs case N (%)	COR [95%CI]	AOR [95%CI]
**Occupation**			
Under age	556 (1.60)	1.00	
Student	60 (0.43)	1.551.19,1.99]	0.84 [0.53 - 1.34]
Farmer	223 (1.76)	3.403.20,4.97]	0.72[0.57 – 0.91]*
Paid job	257 (5.44)	3.112.33,4.14]	1.13 [0.91 – 1.40]
Unemployed	14 (1.33)	2.92 [1.74,4.87]	0.66 [0.36 - 1.20]
Others	45 (13.72)	35.7 [25.38, 50.43]	1.34 [0.92 – 1.94]
**Doing Work related Physical activity**			
Under age	287 (0.84)	1.00	1.00
Sometimes	484 (2.96)	0.24 [0.21,0.28]	0.72 [0.59 - 0.86] **
Most of the time	384 (2.28)	0.17 [0.14,0.19]	0.64 [0.52 - 0.79] **
**Food security access**			
Food secure	565 (1.77)	1.00	1.00
Mildly food insecure	110 (1.51)	1.19 [0.96,1.46]	1.08 [0.86 - 1.34]
Moderately food insecure	296 (1.56)	1.71 [1.49, 1.96]	1.26 [1.07 - 1.48] *
Severely food insecure	136 (2.37)	2.70 [2.28,3.20]	1.71 [1.37 - 2.12] **
**Location of place**			
High land	924 (2.05)	1.00	1.00
Low Land	231 (1.03)	0.96 [0.85, 1.08	1.01 [0.84 - 1.22]
**Marital status**			
Under age	13 (0.06)	1.00	1.00
Marred	655 (3.00)	4.5 [4.29,6.93]	0.86 [0.20 - 3.61]
Single	82 (0.42)	4.07[3.17, 5.21]	0.37[0.09 – 1.57]
Divorced	142 (5.94)	16.1[12.2,21.3]	0.91 [0.21 - 3.88]
Widowed	234 (9.88)	23.9 [18.4, 31.2]	1.07 [0.25 – 4.55]
Separated	29 (2.94)	7.68 [4.94,11.96]	0.69 [0.15 – 3.06]
**Reported history of Injury**			
No	1139 (1.70)	1.00	1.00
Yes	16 (3.92)	1.90 [1.44, 3.82]	2.05 [0.15 – 3.34]

*:- P-value <0.05; **:- P-value <0.001

Other:- house wife and retired

For peer review only - http://bmjopen.bmj.com/site/about/guidelines.xhtml

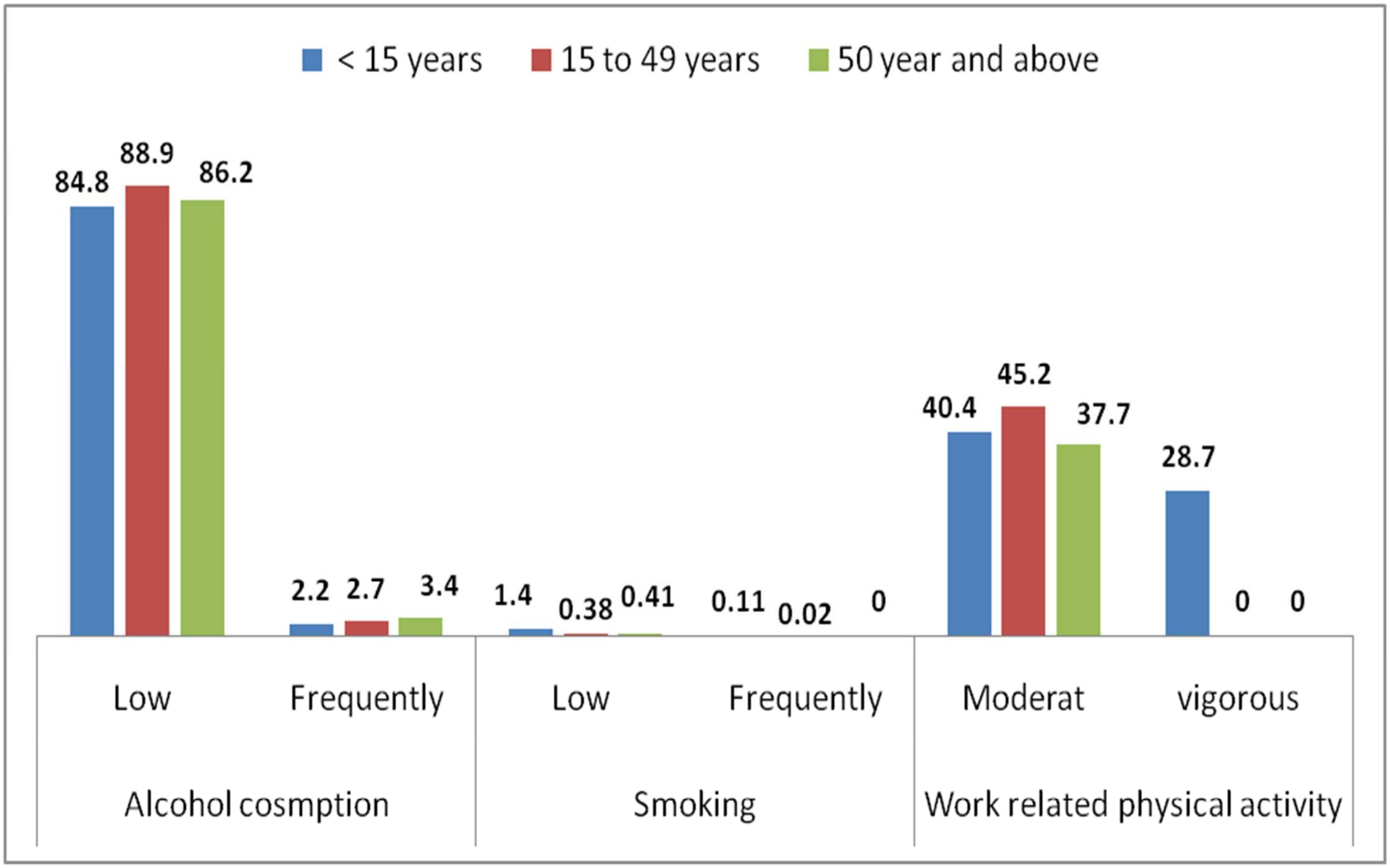

Figure 1: Reported alcohol intake, smoking and physical activity by age group of Dabat HDS research center population, Northwest Ethiopia, 2016

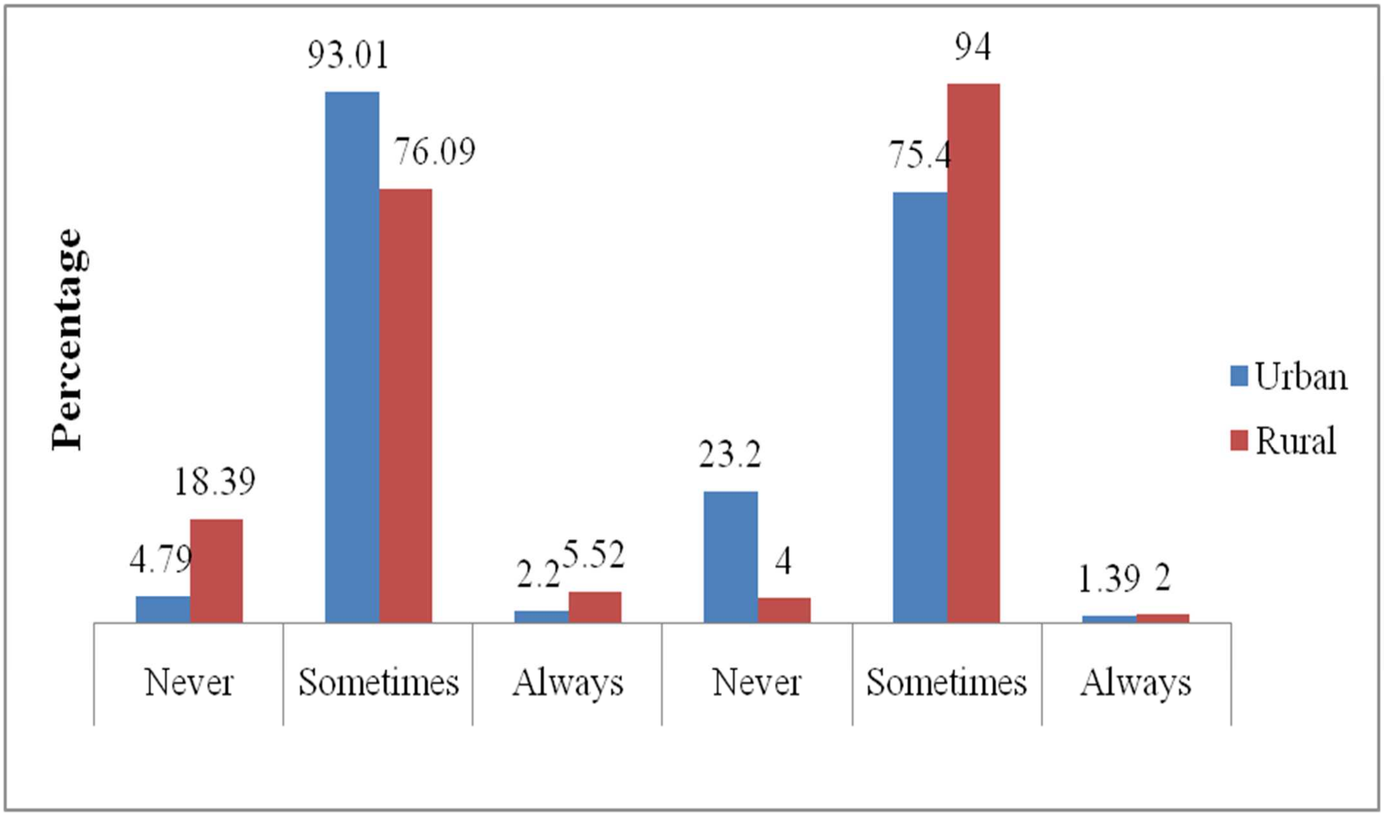

Figure 2: Alcohol consumption of Dabat HDS research center population by alcohol intake, smoking and physical activity, Northwest Ethiopia, 2016

For peer review only

Check list of items that is included in reports of our studies

	Item No.	Page No.
**Title**		**1**
**abstract**	**1**	**2**
**Introduction**		
Background/rationale	2	3
Objectives	3	4
**Methods**		
Study design	4	4
Setting	5	4
Participants	6	5
Data sources/ measurement	7	5
Study size	8	5
Quantitative variables	9	6
Data quality control measures	10	6
Data Analysis Procedures	11	6
Ethical statement	12	7
**Results**		
Participants	13	7
Descriptive data	14	7
Outcome data	15	8
Factors	16	8
**Discussion**		
Key results	17	8
Limitations	18	11
Conclusion	19	11

For peer review only

BMJ Open

The Prevalence of Non-communicable Diseases in Northwest Ethiopia: Survey of Dabat Health and Demographic Surveillance System

Journal:	*BMJ Open*
Manuscript ID	bmjopen-2016-015496.R1
Article Type:	Research
Date Submitted by the Author:	04-May-2017
Complete List of Authors:	Abebe, Solomon; University of Gondar, Institute of Public Health, College of Medicine and Health Science; University of Gondar,<br>Andarge, Gashaw ; University of Gondar, Public Health<br>Shimeka, Alemayehu ; University of Gondar<br>Alemu, Kassahun ; University of Gondar<br>Kebede, Yigzaw ; University of Gonder<br>Wubshet, Mamo; University of Gondar, Public Health<br>Woldemariam, Amare; Human Nutrition<br>Gebeyehu , Abebaw ; University of Gondar<br>Bayisa, Mulugata ; University of Gondar, School of Medicine, College of Medicine and Health Science;<br>Yitayal, Mezgebu ; University of Gondar<br>Awoke, Tadesse; University of Gondar, Epidemiology & Biostatistics<br>Azmeraw,, Temesgen ; University of Gondar<br>Birku, Melkamu ; University of Gondar
**Primary Subject Heading**:	Epidemiology
Secondary Subject Heading:	Cardiovascular medicine, Epidemiology, Public health
Keywords:	NCDs, HDSS, DM

SCHOLARONE™
Manuscripts

For peer review only - http://bmjopen.bmj.com/site/about/guidelines.xhtml

The Prevalence of Non-communicable Diseases in Northwest Ethiopia: Survey of Dabat Health and Demographic Surveillance System

Solomon Mekonnen[1], Gashaw Andargie[2], Alemayehu Shimeka[3], Kassahun Alemu[3], Yigzaw Kebede[3], Mamo Wubeshet[4], Amare Tariku[1], Abebaw Gebeyehu[5], Mulugeta Bayisa[3], Mezgebu Yitayal[2], Tadesse Awoke[3], Temesgen Azmeraw[5], Melkamu Birku[5],

Authors' Information

[1]Department of Human Nutrition, Institute of Public Health, College of Medicine and Health Sciences, University of Gondar, Ethiopia

[2]Department of Health Service Management and Health Economics, Institute of Public Health College of Medicine and Health Sciences, University of Gondar, Ethiopia

[3]Department of Epidemiology and Biostatistics, Institute of Public Health College of Medicine and Health Sciences, University of Gondar, Ethiopia.

[4]Department of Environmental and Occupational Health and Safety, Institute of Public Health College of Medicine and Health Sciences, University of Gondar, Ethiopia

[5]Dabat Research Centre Health and Demographic Surveillance System, Institute of Public Health College of Medicine and Health Sciences, University of Gondar, Ethiopia

[6]Department of Physiotherapy, Institute of Public Health, College of Medicine and Health Sciences, University of Gondar, Ethiopia.

[7]Department of Reproductive Health, Institute of Public Health, College of Medicine and Health Sciences, University of Gondar, Ethiopia.

Authors Email

*SM: solomekonnen@yahoo.com
AS: alemayheushimeka@gmail.com
MB: muluphysio@yahoo.com
AT: amaretariku15@yahoo.com
GA: gashawab@gmail.com
AG: gabebaw2worku@gmail.com
YK: gkyigzaw@yahoo.com
TA:tawoke7@gmail.com
MW:mamo_wubshet@yahoo.com
TA:teme_957@yahoo.com
MB: m.berku@yahoo.com
KA: kassalemu@gmail.com

***Corresponding author**
Solomon Mekonnen
P.o.Box 196, University of Gondar

For peer review only - http://bmjopen.bmj.com/site/about/guidelines.xhtml

1. Abstract

Objective: The main objective of this study was to investigate the magnitude and associated factors of NCDs at the Dabat Health and Demographic Surveillance System (HDSS) site in the north-western part of Ethiopia.

Design: A population-based cross-sectional study was conducted from October to December, 2014

Setting: HDSS site, Ethiopia

Participants: A total population of 67,397 living in 16,053 households was included in the study.

Measures: Structured interviewer-administered questionnaire was used to collect data. Self reported morbidity was used to ascertain NCD. A binary logistic regression model was employed to identify the determinants of NCDs.

Result: One thousand one hundred sixty (1.7%) [95%CI: 1.62, 1.82] participants were found with at least one type of NCD. Heart disease and hypertension which accounted for 404 (32.2%) and 401 (31.9%), of the burden, respectively, were the most commonly reported NCDs, followed by 347 (27.7%) asthma, 62 (4.9%) diabetes mellitus, and 40 (3.2%) cancer cases. Advanced age (≥65 year) (AOR= 19.6; 95% CI: 5.83 65.70), urban residence (AOR= 2.20; 95% CI: 1.83, 2.65) household food insecurity (AOR= 1.71; 95% CI: 1.37, 2.12) and, high income (AOR= 1.28; 95%CI: 1.02, 1.59) were significantly associated with the reported history of NCDs, while low (AOR= 0.36, 95% CI: 0.31, 0.42) and moderate (AOR=0.33 95% CI: 0.22, 0.48) alcohol consumption, farming occupation (AOR= 0.72; 95% CI: 0.57, 0.91), and work related physical activities (AOR= 0.66; 95% CI: 0.50, 0.88) were inversely associated with NCDs.

Conclusion: There is a high burden of NCDs at the Dabat HDS site. Promotion of regular physical exercise and reducing alcohol consumption are essential to mitigate the burden of NCDs. In addition, preventive interventions of NCDs should be strengthened among urban dwellers, older aged and people of higher economic status.

STRENGTHS AND LIMITATIONS OF THE STUDY

- This is the first community-based Prevalence study examining the magnitude and associated factors of NCDs at a health and demographic surveillance site in Ethiopia.
- It has stringently included all age groups
- It deals not only with the prevalence of NCDs but also with factors associated with itOur findings provide important information that can be used to improve screening and care through medical evaluation to detect and manage NCDs.
- One limitation of this study is the use of self-report of NCDs that may introduce recall and social desirability biases and the morbidities reported might not be accurate diagnosis of disease conditions.
- Another limitation is that the study used only a cross-sectional design which might not show temporal relationships; thus, the observed associations might not necessarily be causal.

For peer review only - http://bmjopen.bmj.com/site/about/guidelines.xhtml

Introduction

2. Background

The burden of non-communicable chronic diseases (NCDs) is rising rapidly and has now become a major challenge to global development[1]. Globally, it is the leading cause of death, killing more people than all other causes each year., Low and middle income countries are also disproportionately suffering from the consequences of these diseases [2].

There are significant social, economic, and health losses as a result of the increasing prevalence of NCDs. Even though crises due to NCDs can be deterred through cost-effective and feasible interventions, around 80% of the deaths in these countries are caused by these diseases [3].

Non-communicable diseases have not received equal attention with communicable diseases in middle and low-income countries [4]. There is inadequate information with regard to the epidemiology of NCDs in developing countries for governments to develop NCD related action plans, leaving major policy gaps for intervention. Hence, country-specific epidemiological and experimental studies are required for a better understanding of the burden and causes of such particular NCDs, as diabetes mellitus (DM), cardio vascular diseases (CVD), hypertension(HTN), cancers, and chronic respiratory diseases (CRD) [2].

In Ethiopia, where health outcomes are highly dependent on cultural values, strong research is needed to properly assess lifestyle and anthropological measures to reveal the causes, prevention, and control of NCDs. The country is in a major economic transition, bringing new ways of life to its population. As a result, there is also an epidemiological transition in terms of diseases from infectious to non-communicable diseases attributable to modified risk factors [5]. Some of the modifiable risk factors are physical inactivity, unhealthy diet, increasing alcohol, and tobacco consumption. To halt the increasing prevalence of these diseases, a comprehensive multidisciplinary research is vital to elucidate the root causes and trends in the epidemiological transition

Moreover, the Ethiopian health care system is not designed for chronic health care, whose costs are huge. NCDs add to the nation's burden of poverty, slow down development, increase health inequities, which are believed to impose a huge demand on the healthcare system, creating

For peer review only - http://bmjopen.bmj.com/site/about/guidelines.xhtml

economic pressure in the country. In Ethiopia, NCD prevention and control remains inadequate despite the growing burden of the problem. The few studies conducted in Ethiopia are inconclusive in revealing the epidemiology of the diseases [6]. Besides, almost all of the studies done are institution-based and do not expose the prevalence at the population level. This has contributed to policy gaps, leading to inadequate government investment on prevention and treatment of NCDs [7].

To understand the true burden of NCDs in Ethiopia, population based surveys are essential. Besides, it is time to assess the magnitude of NCDs and its associated factors at the population level for easy and cost-effective prevention.

3. Objective: The main objective of this study was to disclose the magnitude and associated factors of NCDs at a DHSS site in the north-western part of the country.

Methods

4. **Study Design**

A population based cross sectional study was conducted from October to December 2014 at the Dabat HDSS site, Dabat District, northwest Ethiopia.

5. **Setting:** The district is located 820 km from Addis Ababa, the capital of Ethiopia. Based on the 2007 national census conducted by the Central Statistical Agency (CSA) [8], the district has a total projected population of 216,240 in 1,187.93 sq. km of land. Dabat has a population density of 122.49, which is greater than the zonal average of 63.76 persons per sq. km.

   The Dabat HDSS site was launched in the rural district of Dabat, by the Gondar College of Medical and Health Sciences (now University of Gondar) in 1995. In Ethiopia, there is inadequate vital events registration system to date; instead, population and housing census, national sample surveys, and health facility reports are the main sources of information on population health. Thus, Dabat HDS was established in order to produce a longitudinal data to aid in better health policy formulation, informed decision-making and health practice.

For peer review only - http://bmjopen.bmj.com/site/about/guidelines.xhtml

6. **Participants:** At the moment, 67,397 people live in the 16,053 households of the randomly selected 13 (9 rural and 4 urban) kebeles (the lowest administrative units) of the HDSS site. Household level data on the general socio-demography of family members, housing and environmental conditions, food security, chronic diseases, risk factors for non-communicable diseases, injury in the last two weeks, maternal and child health are updated every six months, that is, twice a year. All permanent residents in the DHS area were eligible to participate in the study. The majority of the residents live on subsistence agriculture, producing mainly food crops.
7. **Data sources/ measurement: A** structured, interviewer administered questionnaire was used to collect data on reported history of blood pressure, diabetes, cancer, heart disease, asthma, and history of other risk factors for chronic disease conditions (alcohol consumption, cigarette smoking, vegetable consumption, and physical activity). Data were collected by diploma graduate professionals who were experienced in data collection at the HDS center. The questionnaire was developed by considering questions previously used at this site. The English version of the questionnaire was translated to Amharic and back translated to English by language experts to maintain the consistency of meaning.
8. **Sample size and sampling technique:** All permanent residents (67,397) of the HDSS site living in 16,053 households were included in the study. This study included all the permanent residents at Dabat HDS research center. The heads of households, mostly mothers, were interviewed to capture data on events (NCDs) that happened in the families. The outcome of this study, NCD, was ascertained using reported history of having at least one of the NCDs, such as diabetes mellitus, hypertension, cancer, asthma, and heart disease prior to data collection. Accordingly, a household member was labelled as having non-common chronic diseases if they had at least one reported NCD type. Socio-demographic characteristics (age, sex, ethnicity, religion, marital status, residence, wealth index and family size), food security status, and other health related factors were considered as independent variable.
9. **Quantitative variables:** Food security is defined as existing when "all people, at all times, have physical and economic access to sufficient, safe, and nutritious food to meet their dietary needs and food preferences for an active and healthy life". In order to assess

For peer review only - http://bmjopen.bmj.com/site/about/guidelines.xhtml

the food security status of the households, an 18 item community food insecurity access scale assessment tool was adopted from the Household Food Insecurity Access Scale (HFIAS) Indicator Guide VERSION 3 and categorized into four levels, using the HFIS variables. A frequency of occurrence question was asked to determine whether the condition happened rarely (once or twice), sometimes (three to ten times), or often (more than ten times) in the past four weeks, to find out whether participants were food secure, mildly food insecure, moderately food insecure, or severely food insecure.

Household wealth index was computed for urban and rural residents separately, using the Principal Component Analysis (PCA). Urban wealth status was calculated by considering properties like selected household assets, while only tropical livestock unit was used to determine rural income residents. In the PCA, an Eigen value of greater than one, Kaiser-Mayer-Olkin (KMO) distribution, and in the final model, common factor scores were summed and ranked into lowest, middle, second, fourth, and highest.

10. **Data quality control measures:** The quality of data was insured by pre-testing the data collection tools on 5% of the participants in Debark district which has similar socio-demographic characteristics with Dabat district. Data was collected by 26 diploma graduate professionals and 5 individuals supervised the process. Supervision was also carried out at the spot by the research center leaders. Data collectors and supervisors had a long experience working for the Dabat DHS site. Training was given to both data collectors and supervisors on the questionnaire, content interviewing technique, purpose of the study, and on how to maintain confidentiality of information. The collected data were checked for completeness and clarity by assigned supervisors and the principal investigator.

11. **Data Analysis Procedures:** Data were entered into the HRS 2 software and exported to STATA version 14 for further analysis. The overall prevalence of NCDs was defined as the presence of any of the studied non-communicable chronic diseases during interview among the study population. The results were presented in the form of tables, figures, and summary statistics. Logistic regressions were used to assess the association of various factors and the outcome variable. The Bi-variable analysis with a crude odds ratio of 95% CI was used to see the crude effect of independent variables on NCDs. All independent variables that had a p-value of 0.20 in the Bi-variable analysis were fitted to the multivariable logistic regression analysis to control for possible confounding effects of

For peer review only - http://bmjopen.bmj.com/site/about/guidelines.xhtml

variables. Variables which were found to be significant at a p-value 0.05 level and 95% CI were considered to be the determinant factors of NCDs.

12. **Ethical considerations**: Ethical clearance was obtained from the Institutional Review Board (IRB) of the University of Gondar before starting the actual data collection. Participants in the selected kebeles were recruited voluntarily after obtaining full information about the research and signed a written consent agreement. They were informed of their rights to withdraw from the study at any stage. Moreover, the confidentiality of information was guaranteed by using code numbers rather than personal identifiers and by keeping the data locked. . Individuals identified as cases of NCDs were referred to the nearby clinic for further treatment and follow-up.

For peer review only

Results

13. **Participants:** A total of 67,397 people were enrolled in this study. Women comprised about 50.77%. The majority (75.10%) of the study population was lived in areas. Among the study subjects, 42.96% (28,952) were under 15 years of age. Christianity is the dominant religion, accounting for 96.36%of the participants. The socio-demographic characteristics of the study population are depicted in Table 1.

14. **Descriptive data:** About 8.87% of the participants never drank alcohol in their life, while 88.2% and 2.88% were occasional and frequent consumers, respectively. About 99.5% of the study participants never smoked cigarettes in their lifetime, and 44.7% performed vigorous work related physical activities on daily bases (Fig1 and 2).

    Figure 1: Reported alcohol intake, smoking and physical activity by age group of Dabat HDS research center population, Northwest Ethiopia, 2016

    Figure 2: Frequency smoking, physical activity, and alcohol consumption of Dabat HDSS research center population, north-west Ethiopia, 2016

15. **Outcome data:** Participants who had NCDs were 1160 (1.7%) [95%CI: 1.62, 1.82], and according to the proportionate NCDs, morbidity ratio (morbidity fraction) was 404 (32.2%) heart disease, 401 (31.9%) hypertension, 347 (27.7%) asthma, 62 (4.9%) diabetes mellitus, and 40 (3.2%) cancer (Table 2). The mean (±SD) age of subjects with NCDs was 49.32 (±17.9). Out of the 16,035 households, 1146 (6.98%) had at least one

For peer review only - http://bmjopen.bmj.com/site/about/guidelines.xhtml

NCD per household in the HDSS, and 40 (3.6%) of these had two NCDs cases per household. Of these, 746 (64.6%) were female with 48.96% in food insecure group. The prevalence of all types NCDs was slightly higher among women in all age groups.

16. **Factors:** After adjusting for a number of important covariates, the multivariable logistic regression analysis showed that increasing age, alcohol consumption, high income, living in urban areas, farming, work related physical activities, and severe food insecurity were associated with the odds of NCDs. Advanced age (≥ 65 year) (AOR= 19.6; 95% CI: 5.83 – 65.7), living in urban areas (AOR= 2.20; 95% CI: 1.83, 2.65), and severe household food insecurity (AOR= 1.71; 95% CI: 1.37, 2.12) were significantly associated with reported history of NCDs, while low and moderate alcohol consumption (AOR= 0.36, 95% CI: 0.31, 0.42) and (AOR=0.33 95% CI: 0.22, 0.48), high income (AOR= 1.28; 95%CI: 1.02, 1.59), farming occupation (AOR= 0.72; 95% CI: 0.57, 0.91), and work related physical activities (AOR= 0.66; 95% CI: 0.50, 0.88) were inversely associated with NCDs (Table 3).

Discussion

Key result: The number of NCD cases seen at the Dabat HDS site was larger among the elderly; the proportions were considerably high among urban dwellers. A similar to another study (9) done in rural and urban Ethiopia, older age, physical inactivity, living in urban areas, farming, alcohol consumption, severe food insecurity, and low income were the strongest predictors of NCDs. [9]. Similar review study done in Sub-Saharan countries showed that NCDs are now the second most common causes of death after infectious diseases [10]. Our findings show that the occurrence of NCDs is significantly high among severely food insecure groups. This study is supported by a finding from Bangladesh which showed that persons with shorter stature are more likely to develop NCDs like diabetes and hypertension [11]. The WHO disease classification report shows that the disease phenotype encountered in Africa is very different from the West and most closely resembles malnutrition-related. Some researchers also indicated that early exposure to cereal products or solid foods might increase immune response, which could trigger the destruction of β-cells[12]. Another finding shows that people who were small

For peer review only - http://bmjopen.bmj.com/site/about/guidelines.xhtml

at birth and grew least during the first year of life had a chance of developing NCDs in later life [13]. This disease burden can affect the normal growth of the people, there-by developing early onset of non-communicable diseases in early-life and childhood. This exposure is likely to be responsible for the observed high rate of prevalence in this low economic status group [14]. Likewise, in our study a high level of NCDs were noted among individuals with high income.

This can be plausibly explained by the fact that people with better economic status will be more likely to favour mechanization which in turn leads to transition from physically active to less physically active sedentary lifestyle, keeping people indoors. Moreover, people with better economic status will be more likely to follow unhealthy eating habits as they can afford diets richer in un-saturated fat, thereby being more susceptible to obesity and development of NCDs. [12, 15]. These data suggest that the burden of NCDs is increasing, perhaps as a result of demographic and epidemiological changes. Even though the country is undergoing a delayed transition, the burden of both malnutrition related and non-communicable diseases remains high [16, 17]. This double burden has a negative implication on major economic transitions [18].

The effect of ageing on NCD prevalence is already evident in Africa [19]. Aging causes a progressive decline in the strength of musculature, which causes muscle atrophy and the risk of developing CVD. In our finding, the prevalence of NCDs increases with advancing age. This finding is consistent with other reports from India, [12] as NCD epidemic matures, the onset will shift to younger age-groups. At aging, many more people are exposed to the risk factors for long periods until the complications develop and they experience the clinical syndromes of NCDs [16]. In Ethiopia, lack of awareness, distance to facilities, and un affordability of health services delay diagnoses for the elderly
[20].

For peer review only - http://bmjopen.bmj.com/site/about/guidelines.xhtml

Physical activity is more common in rural than urban regions of Africa because rural dwellers often rely on intense agricultural activities as their main occupation. In line with this, our finding shows that the prevalence of NCDs is low among farmers and physically active individuals which is consistent with the meta-analysis done on the risk factors for chronic non-communicable diseases in China, Ghana, Mexico, India, Russia, and South Africa[21]. Sedentary lifestyle and lack of physical activities accelerate the occurrence of NCDs; this condition is a growing challenge to sub-Saharan countries [22, 23]. Walking time and space are drastically reduced in an urban community as compared with a rural community. This reduction in physical activity associated with city life, partly explains the excessive prevalence of NCDs in urban areas. Studies show that lack of physical activity appears to be a significant risk factor for CVD in Sub-Saharan Africa [12, 16]. Reductions in physical activity and more sedentary modes of living parallel nutrition transition, producing an overall positive energy balance resulting in a paradigm of lifestyle transition [24].

In developing countries, rapid and unplanned urbanization and modernization resulting in socio-economic and behavioural changes in people directly affects the health of the urban population [16, 25]. The main difference in physical activity between the two types of community, however, is the use of walking in rural areas as means of transportation. A diet high in total fat, cholesterol, sugar and other refined carbohydrates, and low in polyunsaturated fatty acids and fibre, often accompanied by an increasingly sedentary life, characterize life in the urban community [25]. In other presumptions people exposed to periodic famines, which frequently occur in Africa, would through natural selection increase the frequency of certain genetic trait(s); these genes, also called “thrifty genes,” will be expressed as individuals get exposed to undernutrition and starvation. This adaptation will enable individuals be able to cope with the shortage of energy by supressing body metabolism and limiting it only to sustaining the highly vital metabolisms of the body. However, when such individuals with activated thrifty genes from undernutrition experience improvement in their nutritional intake, the already supressed level of body metabolism may not be able to improve to metabolize the otherwise normal nutritional intake. This inability to sufficiently metabolize nutrients

For peer review only - http://bmjopen.bmj.com/site/about/guidelines.xhtml

will lead to fat accumulation, dyslipidaemia, and diabetes despite the individual's relatively normal nutritional intake. Therefore, a continued discussion of proven cost-effective interventions of non-communicable chronic diseases, will be useful for improving public health policy [24].

Strengths of the study: This is the first community-based study examining the magnitude and associated factors of NCDs in the wider population, HDSS site, in Ethiopia.

- The findings of this study have provided important information that can be used to improve screening and care through medical evaluation to detect and manage NCDs.

17. **Limitations:** The limitation of this study is that it has used self-reported NCDs that may introduce recall and social desirability biases, and the morbidities reported might not be accurate diagnosis of the disease condition, because diseases might be miss-classified. Moreover, since this re-census is a general survey of the population, it may not provide in-depth information about specific diseases which have occurred in the community. Another limitation of this study is that it used only a cross-sectional design which might not show temporal relationships; thus, the observed associations might not necessarily be causal. Therefore, a longitudinal research is needed to assess the relationships among the variables over time.

18. Conclusion: The number of NCD cases seen at the Dabat HDS site is higher among the elderly, and the proportions were considerably higher among the urban population. Effective and sustainable strategy for the prevention of chronic diseases throughout, implementing new intervention strategies by providing playgrounds to the urban community, improving the lively-hood of the rural community, and increasing access to healthy foods are highly essential.

Acknowledgement

We would like to acknowledge the University of Gondar and EPH CDC for the financial support. We extend our thanks to the Dabat District Health Office for their permission to conduct the study. We want to express our gratitude to all staff of the Dabat DHS and the study participants.

For peer review only - http://bmjopen.bmj.com/site/about/guidelines.xhtml

Authors' contributions

SM, GA, AG, YK, TA, MW, TA, MB, KA designed the study, SM, AS, MB, AT performed the analysis and interpretation of data and drafted the paper. SM, AS, MB, AT SM, GA, AG, YK, TA, MW, TA, MB, KA, revised the drafts of the paper. All authors prepared the draft manuscript, read and approved the final manuscript.

Funding: There are no funders to report for this submission

The authors declare no competing interests.

Data sharing statement: No additional data are available.

References

1. Habib SH, S.S., *Burden of non-communicable disease: Global overview. .* Diabetes Metab Syndr Clin Res Rev. , 2010. **4** (1): p. 41-47.
2. Miranda, J.J., et al., *Non-communicable diseases in low- and middle-income countries: context, determinants and health policy.* Trop Med Int Health, 2008. **13**(10): p. 1225-34.
3. Kankeu, H.T., et al., *The financial burden from non-communicable diseases in low- and middle-income countries: a literature review.* Health Res Policy Syst, 2013. **11**: p. 31.
4. Boutayeb, A., *The double burden of communicable and non-communicable diseases in developing countries.* Trans R Soc Trop Med Hyg, 2006. **100**(3): p. 191-9.
5. Lee, I.M., et al., *Effect of physical inactivity on major non-communicable diseases worldwide: an analysis of burden of disease and life expectancy.* Lancet, 2012. **380**(9838): p. 219-29.
6. Mulu, A., et al., *Epidemiological and clinical correlates of malaria-helminth co-infections in Southern Ethiopia.* Malar J, 2013. **12**: p. 227.
7. Murray, C.J. and A.D. Lopez, *Global mortality, disability, and the contribution of risk factors: Global Burden of Disease Study.* Lancet, 1997. **349**(9063): p. 1436-42.
8. *Summary and statistical report of the 2007 population and housing census, Ethiopia* in *(CSA).* 2007.

For peer review only - http://bmjopen.bmj.com/site/about/guidelines.xhtml

9. Tesfaye, F., *Epidemiology of Cardiovascular Disease Risk Factors in Ethiopia The rural-urban gradient* in *Department of Public Health and Clinical Medicine*. 2008, Umeå University: Umeå University, Umeå 2008:2004042.
10. Mbewu, A. and J.C. Mbanya, *Cardiovascular Disease*, in *Disease and Mortality in Sub-Saharan Africa*, D.T. Jamison, et al., Editors. 2006: Washington (DC).
11. Hoque, M.E., M.R. Khokan, and W. Bari, *"Impact of stature on non-communicable diseases: evidence based on Bangladesh Demographic and Health Survey, 2011 data".* BMC Public Health, 2014. **14**: p. 1007.
12. Yoon, J., et al., *The Non-Communicable Disease Burden in Korea: Findings from the 2012 Korean Burden of Disease Study.* J Korean Med Sci, 2016. **31 Suppl 2**: p. S158-S167.
13. Martyn, C.N. and D.J. Barker, *Reduced fetal growth increases risk of cardiovascular disease.* Health Rep, 1994. **6**(1): p. 45-53.
14. Seligman, H.K., et al., *Food insecurity and glycemic control among low-income patients with type 2 diabetes.* Diabetes Care, 2012. **35**(2): p. 233-8.
15. Mbanya, J.C., et al., *Diabetes in sub-Saharan Africa.* Lancet, 2010. **375**(9733): p. 2254-66.
16. Steyn, K. and A. Damasceno, *Lifestyle and Related Risk Factors for Chronic Diseases*, in *Disease and Mortality in Sub-Saharan Africa*, D.T. Jamison, et al., Editors. 2006: Washington (DC).
17. Adetunji, J. and E.R. Bos, *Levels and Trends in Mortality in Sub-Saharan Africa: An Overview*, in *Disease and Mortality in Sub-Saharan Africa*, D.T. Jamison, et al., Editors. 2006: Washington (DC).
18. Musaiger, A.O. and H.M. Al-Hazzaa, *Prevalence and risk factors associated with nutrition-related noncommunicable diseases in the Eastern Mediterranean region.* Int J Gen Med, 2012. **5**: p. 199-217.
19. Ayernor, P.K., *Diseases of ageing in Ghana.* Ghana Med J, 2012. **46**(2 Suppl): p. 18-22.
20. Sikder, S.S., et al., *Care-seeking patterns for fatal non-communicable diseases among women of reproductive age in rural northwest Bangladesh.* BMC Womens Health, 2012. **12**: p. 23.
21. Wu, F., et al., *Common risk factors for chronic non-communicable diseases among older adults in China, Ghana, Mexico, India, Russia and South Africa: the study on global AGEing and adult health (SAGE) wave 1.* BMC Public Health, 2015. **15**: p. 88.
22. Thornton, J.S., et al., *Physical Activity Prescription: A Critical Opportunity to Address a Modifiable Risk Factor for the Prevention and Management of Chronic Disease: A Position Statement by the Canadian Academy of Sport and Exercise Medicine.* Clin J Sport Med, 2016. **26**(4): p. 259-65.

For peer review only - http://bmjopen.bmj.com/site/about/guidelines.xhtml

23. Mbanya, J.C. and K. Ramiaya, *Diabetes Mellitus*, in *Disease and Mortality in Sub-Saharan Africa*, D.T. Jamison, et al., Editors. 2006: Washington (DC).

24. Zaman, M.M., et al., *Prevalence of risk factors for non-communicable diseases in Bangladesh: Results from STEPS survey 2010.* Indian J Public Health, 2016. **60**(1): p. 17-25.

25. C. Angkurawaranon, W.J., B. Chenthanakij, P. Doyle, D. Nitsch, *Urbanization and non-communicable disease in Southeast Asia: a review of current evidence.* Public health, October 2014. **128**(10): p. 886-895.

For peer review only

For peer review only - http://bmjopen.bmj.com/site/about/guidelines.xhtml

Table 1: Socio-demographic characteristics of the population of Dabat HDS research center, Northwest Ethiopia, 2016.

Variables	Frequency	Percent
Sex Male	33,181	49.23
Female	34,214	50.77
Age in years		
Under 15	28,952	42.96
15 to 24	13,289	19.72
25 to 34	8,170	12.12
35 to 44	6,322	9.38
45 to54	4,720	7.00
55 to 64	3,020	4.48
65 and above	2,918	4.33
Residence		
Rural	50,769	75.10
Urban	16,835	16.84
Marital status		
Under 10 years old	20,089	29.81
Married	21,814	32.37
Single	19,746	29.30
Divorced	2,390	3.55
Widowed	2,369	3.52
Separated	917	1.36
Cohabiting	68	0.10
Religion		
Orthodox	64,940	96.36
Muslim	2,444	3.63
Others (Catholic, Protestant)	11	0.01
Ethnicity		
Amhara	67,294	99.85
Tigre	84	0.12
Others (Oromo,Agaw)	17	0.02
Education		
Not on education (<7years)	13,672	20.29
Unable to read and write	21,149	31.38
Read and write	5,541	8.22
Grade 1-4	10,960	16.26
Grade 5-6	4,560	6.77
Grade 7-8	3,590	5.33
Grade 9-10	4,375	6.49
Grade 11-12	1,957	2.90
Grade 12 and above	1,591	2.36

For peer review only - http://bmjopen.bmj.com/site/about/guidelines.xhtml

For peer review only

Variables	Frequency	Percent
Occupation		
No occupation(under 10 years)	20,436	38.45
Students	13,955	26.26
Farmers	12,647	23.80
Employed permanent	1,951	3.67
Private job	1,167	2.20
Job seeker	1056	1.99
Merchant	656	1.23
House maid	623	1.17
Employed contract	328	0.62
Retired	296	0.56
Others(housewife, shepherd, disabled)	33	0.05
Location		
Low land	22,380	66.9
High land	45,224	33.1
Relation to the HH head		
HH head	16,082	23.86
Housewife	10,542	15.64
Son/daughter	34,702	51.49
Sister/brother	538	0.80
Mother/father	310	0.46
Grandson/granddaughter	3095	4.59
Other relative	1196	1.77
Other non-relative	930	1.38
Family size		
1-4	24,512	36.35
5-9	41,667	61.79
10-15	1,250	1.85
Wealth status		
Poorest quintile	9,475	14.58
Second quintile	11,344	17.45
Third quintile	13,031	20.05
Fourth quintile	14,593	22.45
Richest Quintile	16,577	25.47

For peer review only

For peer review only - http://bmjopen.bmj.com/site/about/guidelines.xhtml

Table 2: Proportion of Chronic Non-Communicable Diseases by age, and sex among Dabat HDS sit population, Northwest Ethiopia, 2016.

Variable	Gender	Reported cases age in Year n(%)						
		**1-14**	**15-24**	**25-34**	**35-44**	**45-54**	**55-64**	**≥ 65**
Diabetes	Female	1(0.01)	5 (0.08)	2 (0.05)	3(0.09)	3 (0.12)	7 (0.45)	5 (0.38)
mellitus	Male	4 (0.03)	7 (0.10)	7 (0.18)	5 (0.17)	4 (0.19)	4 (0.28)	5 (0.31)
Hypertension	Female	1 (0.010)	6(0.09)	22 (0.50)	30 (0.91)	49(1.90)	67 (4.26)	86 (6.56)
	Male	2 (0.01)	4 (0.06)	3 (0.08)	15 (0.50)	24 (1.12)	29 (2.0)	60 (3.73)
Heart Disease	Female	10 (0.07)	31(0.47)	54 (1.24)	50 (1.52)	66 (2.56)	43 (2.74)	47 (3.59)
	Male	4 (0.030)	12 (0.18)	14 (0.37)	14 (0.46)	19 (0.89)	12 (0.83)	27 (1.68)
Asthma	Female	2(0.01)	7(0.11)	32 (0.73)	41(1.24)	44(1.71)	36(2.29)	31(2.37)
	Male	4(0.03)	6(0.09)	21(0.55)	33(1.09)	32(1.50)	29 (2.0)	28(1.74)
Mental health	Female	12 (0.08)	26 (0.39)	27 (0.62)	18 (0.55)	19 (0.74)	11(0.70)	9(0.69)
	Male	11 (0.08)	21 (0.31)	16 (0.42)	14 (0.46)	16 (0.75)	3 (0.21)	5 (0.31)
Cancer	Female	2 (0.01)	0 (0.00)	2 (0.05)	6 (0.18)	10 (0.39)	0 (0.00)	6 (0.46)
	Male	1 (0.01)	2(0.03)	1(0.03)	1(0.03)	1 (0.05)	5 (0.35)	3 (0.19)

For peer review only

For peer review only - http://bmjopen.bmj.com/site/about/guidelines.xhtml

Table 3: Factors associated with NCDs of the population of Dabat HDSS research center, Northwest Ethiopia, 2016.

Variables	NCDs case n(%)	COR [95%CI]	AOR [95%CI]
**Sex**			
Female	746 (2.18)	1.00	1.00
Male	409 (1.23)	0.83 [0.74,0.93]	0.85 [0.69 - 1.03]
**Age in year**			
0 to 14	**30 (0.10)**	**1.00**	1.00
15 to 24	78 (0.59)	2.76 [2.20,3.45]	1.55 [0.47, 5.10]
25 to34	153 (1.87)	4.17 [3.32, 5.23]	3.64[1.09- 12.13] *
35 to44	188 (2.97)	4.73 [3.73,5.99]	6.33 [1.89- 21.2]**
45 to54	233 (4.94)	7.23 [5.74,9.12]	11.2 [3.36- 37.5] **
55 to64	209 (6.92)	10.1 [7.92,12.79]	16.1[4.79 - 53.8] **
≥ 65	264 (9.05)	26.27 [21.2,32.2]	19.6 [5.83 – 65.7] **
**Alcohol consumption**			
Never	340 (1.03)	1.00	1.00
Occasional	782 (2.35)	0.50 [0.42, 0.59]	0.36 [0.31 - 0.42] **
2 to 3 times a week	33 (3.04)	0.47 [0.31, 0.71]	0.33 [0.22 - 0.48] **
**Smoking status**			
No	1,110 (2.96)	1.00	1.00
Yes	6 (3.51)	1.73 [0.85, 3.52]	0.80 [0.34 - 1.91]
**Wealth status**			
Poor income	170 (1.80)	1.00	1.00
Low income	172 (1.52)	0.67 [0.57, 0.79]	0.90 [0.71 - 1.14]
Mid-level income	175 (1.34)	0.45 [0.38,0.55]	0.91 [0.72 - 1.16]
Better income	279 (1.91)	0.48 [0.41,0.57]	1.19 [0.96 – 1.49]
High income	336 (2.03)	0.35 [0.29,0.42]	1.28 [1.02 - 1.59] *
**Residence**			
Rural	538 (1.06)	1.00	1.00
Urban	617 (3.68)	1.06 [0.93,1.20]	2.20 [1.83 - 2.65] **
**Education status**			
Illiterate	10 (0.07)	1.00	1.00
Can read &write	646 (3.05)	12.549.25,16.99]	0.85 [0.20 - 3.52]
Primary school	140 (2.53)	6.274.41,8.91]	0.87 [0.21 – 3.61]
High school	55 (0.50)	2.48 [1.72, 3.56]	1.29 [0.31 – 5.39]
Diploma and above	304 (1.89)	3.15 [2.25, 4.40]	1.43 [0.35 – 5.89]

For peer review only - http://bmjopen.bmj.com/site/about/guidelines.xhtml

VARIABLES	NCDs case N (%)	COR [95%CI]	AOR [95%CI]
**Occupation**			
Under age	556 (1.60)	1.00	
Student	60 (0.43)	1.551.19,1.99]	0.84 [0.53 - 1.34]
Farmer	223 (1.76)	3.403.20,4.97]	0.72[0.57 – 0.91]*
Paid job	257 (5.44)	3.112.33,4.14]	1.13 [0.91 – 1.40]
Unemployed	14 (1.33)	2.92 [1.74,4.87]	0.66 [0.36 - 1.20]
Others	45 (13.72)	35.7 [25.38, 50.43]	1.34 [0.92 – 1.94]
**Doing Work related Physical activity**			
Under age	287 (0.84)	1.00	1.00
Sometimes	484 (2.96)	0.24 [0.21,0.28]	0.72 [0.59 - 0.86] **
Most of the time	384 (2.28)	0.17 [0.14,0.19]	0.64 [0.52 - 0.79] **
**Food security access**			
Food secure	565 (1.77)	1.00	1.00
Mildly food insecure	110 (1.51)	1.19 [0.96,1.46]	1.08 [0.86 - 1.34]
Moderately food insecure	296 (1.56)	1.71 [1.49, 1.96]	1.26 [1.07 - 1.48] *
Severely food insecure	136 (2.37)	2.70 [2.28,3.20]	1.71 [1.37 - 2.12] **
**Location of place**			
High land	924 (2.05)	1.00	1.00
Low Land	231 (1.03)	0.96 [0.85, 1.08	1.01 [0.84 - 1.22]
**Marital status**			
Under age	13 (0.06)	1.00	1.00
Marred	655 (3.00)	4.5 [4.29,6.93]	0.86 [0.20 - 3.61]
Single	82 (0.42)	4.07[3.17, 5.21]	0.37[0.09 – 1.57]
Divorced	142 (5.94)	16.1[12.2,21.3]	0.91 [0.21 - 3.88]
Widowed	234 (9.88)	23.9 [18.4, 31.2]	1.07 [0.25 – 4.55]
Separated	29 (2.94)	7.68 [4.94,11.96]	0.69 [0.15 – 3.06]
**Reported history of Injury**			
No	1139 (1.70)	1.00	1.00
Yes	16 (3.92)	1.90 [1.44, 3.82]	2.05 [0.15 – 3.34]

*:- P-value <0.05; **:- P-value <0.001

Other: - house wife and retired

For peer review only - http://bmjopen.bmj.com/site/about/guidelines.xhtml

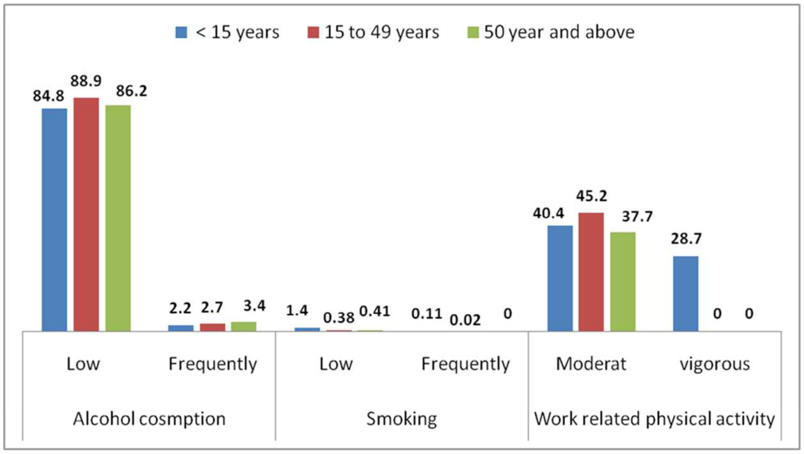

279x215mm (300 x 300 DPI)

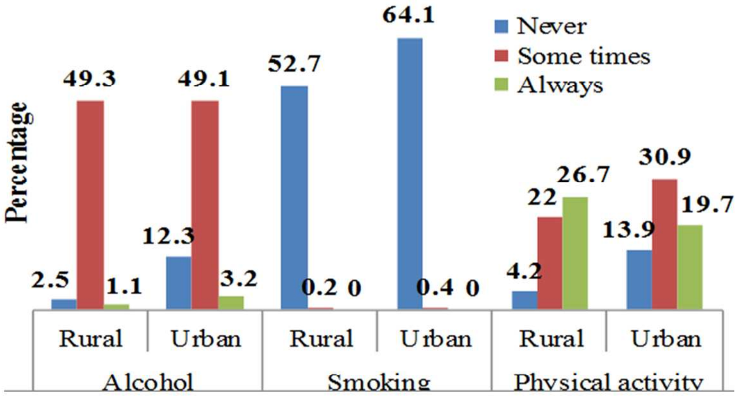

279x215mm (300 x 300 DPI)

Check list of items that is included in reports of our studies

	Item No.	Page No.
**Title**		**1**
**abstract**	**1**	**2**
**Introduction**		
Background/rationale	2	3
Objectives	3	4
**Methods**		
Study design	4	4
Setting	5	4
Participants	6	5
Data sources/ measurement	7	5
Study size	8	5
Quantitative variables	9	6
Data quality control measures	10	6
Data Analysis Procedures	11	6
Ethical statement	12	7
**Results**		
Participants	13	7
Descriptive data	14	7
Outcome data	15	8
Factors	16	8
**Discussion**		
Key results	17	8
Limitations	18	11
Conclusion	19	11

For peer review only

The Prevalence of Non-communicable Diseases in Northwest Ethiopia: Survey of Dabat Health and Demographic Surveillance System

Journal:	*BMJ Open*
Manuscript ID	bmjopen-2016-015496.R2
Article Type:	Research
Date Submitted by the Author:	27-May-2017

Complete List of Authors:	Abebe, Solomon; University of Gondar, Institute of Public Health, College of Medicine and Health Science; University of Gondar,<br>Andarge, Gashaw ; University of Gondar, Public Health<br>Shimeka, Alemayehu ; University of Gondar<br>Alemu, Kassahun ; University of Gondar<br>Kebede, Yigzaw ; University of Gonder<br>Wubshet, Mamo; University of Gondar, Public Health<br>Woldemariam, Amare; Human Nutrition<br>Gebeyehu , Abebaw ; University of Gondar<br>Bayisa, Mulugata ; University of Gondar, School of Medicine, College of Medicine and Health Science;<br>Yitayal, Mezgebu ; University of Gondar<br>Awoke, Tadesse; University of Gondar, Epidemiology & Biostatistics<br>Azmeraw,, Temesgen ; University of Gondar<br>Birku, Melkamu ; University of Gondar

**Primary Subject Heading**:	Epidemiology
Secondary Subject Heading:	Cardiovascular medicine, Epidemiology, Public health
Keywords:	Diabetic foot < DIABETES & ENDOCRINOLOGY, Cardiac Epidemiology < CARDIOLOGY, Hypertension < CARDIOLOGY

For peer review only - http://bmjopen.bmj.com/site/about/guidelines.xhtml

The Prevalence of Non-communicable Diseases in Northwest Ethiopia: Survey of Dabat Health and Demographic Surveillance System

Solomon Mekonnen[1], Gashaw Andargie[2], Alemayehu Shimeka[3], Kassahun Alemu[3], Yigzaw Kebede[3], Mamo Wubeshet[4,] Amare Tariku[1,] Abebaw Gebeyehu[5,] Mulugeta Bayisa[3], Mezgebu Yitayal[2], Tadesse Awoke[3], Temesgen Azmeraw[5], Melkamu Birku[5],

Authors' Information

[1]Department of Human Nutrition, Institute of Public Health, College of Medicine and Health Sciences, University of Gondar, Ethiopia

[2]Department of Health Service Management and Health Economics, Institute of Public Health College of Medicine and Health Sciences, University of Gondar, Ethiopia

[3]Department of Epidemiology and Biostatistics, Institute of Public Health College of Medicine and Health Sciences, University of Gondar, Ethiopia.

[4]Department of Environmental and Occupational Health and Safety, Institute of Public Health College of Medicine and Health Sciences, University of Gondar, Ethiopia

[5]Dabat Research Centre Health and Demographic Surveillance System, Institute of Public Health College of Medicine and Health Sciences, University of Gondar, Ethiopia

[6]Department of Physiotherapy, Institute of Public Health, College of Medicine and Health Sciences, University of Gondar, Ethiopia.

[7]Department of Reproductive Health, Institute of Public Health, College of Medicine and Health Sciences, University of Gondar, Ethiopia.

Authors Email

*SM: solomekonnen@yahoo.com
AS: alemayheushimeka@gmail.com
MB: muluphysio@yahoo.com
AT: amaretariku15@yahoo.com
GA: gashawab@gmail.com
AG: gabebaw2worku@gmail.com
YK: gkyigzaw@yahoo.com
TA:tawoke7@gmail.com
MW:mamo_wubshet@yahoo.com
TA:teme_957@yahoo.com
MB: m.berku@yahoo.com
KA: kassalemu@gmail.com

***Corresponding author**

Solomon Mekonnen
P.o.Box 196, University of Gondar

For peer review only - http://bmjopen.bmj.com/site/about/guidelines.xhtml

1. Abstract

Objective: The main objective of this study was to investigate the magnitude and associated factors of NCDs at the Dabat Health and Demographic Surveillance System (HDSS) site in the north-western part of Ethiopia.

Design: A population-based cross-sectional study was conducted from October to December, 2014

Setting: HDSS site, Ethiopia

Participants: A total population of 67,397 living in 16,053 households was included in the study.

Measures: Structured interviewer-administered questionnaire was used to collect data. Self reported morbidity was used to ascertain NCD. A binary logistic regression model was employed to identify the determinants of NCDs.

Result: One thousand one hundred sixty (1.7%) [95%CI: 1.62, 1.82] participants were found with at least one type of NCD. Heart disease and hypertension which accounted for 404 (32.2%) and 401 (31.9%), of the burden, respectively, were the most commonly reported NCDs, followed by 347 (27.7%) asthma, 62 (4.9%) diabetes mellitus, and 40 (3.2%) cancer cases. Advanced age (≥65 year) (AOR= 19.6; 95% CI: 5.83 65.70), urban residence (AOR= 2.20; 95% CI: 1.83, 2.65) household food insecurity (AOR= 1.71; 95% CI: 1.37, 2.12) and, high income (AOR= 1.28; 95%CI: 1.02, 1.59) were significantly associated with the reported history of NCDs, while low (AOR= 0.36, 95% CI: 0.31, 0.42) and moderate (AOR=0.33 95% CI: 0.22, 0.48) alcohol consumption, farming occupation (AOR= 0.72; 95% CI: 0.57, 0.91), and work related physical activities (AOR= 0.66; 95% CI: 0.50, 0.88) were inversely associated with NCDs.

Conclusion: There is a high burden of NCDs at the Dabat HDS site. Promotion of regular physical exercise and reducing alcohol consumption are essential to mitigate the burden of NCDs. In addition, preventive interventions of NCDs should be strengthened among urban dwellers, older aged and people of higher economic status.

For peer review only - http://bmjopen.bmj.com/site/about/guidelines.xhtml

STRENGTHS AND LIMITATIONS OF THE STUDY

- This is the first community-based Prevalence study examining the magnitude and associated factors of NCDs at a health and demographic surveillance site in Ethiopia.
- It has stringently included all age groups
- It deals not only with the prevalence of NCDs but also with factors associated with itOur findings provide important information that can be used to improve screening and care through medical evaluation to detect and manage NCDs.
- One limitation of this study is the use of self-report of NCDs that may introduce recall and social desirability biases and the morbidities reported might not be accurate diagnosis of disease conditions.
- Another limitation is that the study used only a cross-sectional design which might not show temporal relationships; thus, the observed associations might not necessarily be causal.

Introduction

2. Background

The burden of non-communicable chronic diseases (NCDs) is rising rapidly and has now become a major challenge to global development[1]. Globally, it is the leading cause of death, killing more people than all other causes each year., Low and middle income countries are also disproportionately suffering from the consequences of these diseases [2].

There are significant social, economic, and health losses as a result of the increasing prevalence of NCDs. Even though crises due to NCDs can be deterred through cost-effective and feasible interventions, around 80% of the deaths in these countries are caused by these diseases [3].

Non-communicable diseases have not received equal attention with communicable diseases in middle and low-income countries [4]. There is inadequate information with regard to the epidemiology of NCDs in developing countries for governments to develop NCD related action plans, leaving major policy gaps for intervention. Hence, country-specific epidemiological and experimental studies are required for a better understanding of the burden and causes of such

For peer review only - http://bmjopen.bmj.com/site/about/guidelines.xhtml

particular NCDs, as diabetes mellitus (DM), cardio vascular diseases (CVD), hypertension(HTN), cancers, and chronic respiratory diseases (CRD) [2].

In Ethiopia, where health outcomes are highly dependent on cultural values, strong research is needed to properly assess lifestyle and anthropological measures to reveal the causes, prevention, and control of NCDs. The country is in a major economic transition, bringing new ways of life to its population. As a result, there is also an epidemiological transition in terms of diseases from infectious to non-communicable diseases attributable to modified risk factors [5]. Some of the modifiable risk factors are physical inactivity, unhealthy diet, increasing alcohol, and tobacco consumption. To halt the increasing prevalence of these diseases, a comprehensive multidisciplinary research is vital to elucidate the root causes and trends in the epidemiological transition

Moreover, the Ethiopian health care system is not designed for chronic health care, whose costs are huge. NCDs add to the nation's burden of poverty, slow down development, increase health inequities, which are believed to impose a huge demand on the healthcare system, creating economic pressure in the country. In Ethiopia, NCD prevention and control remains inadequate despite the growing burden of the problem. The few studies conducted in Ethiopia are inconclusive in revealing the epidemiology of the diseases [6]. Besides, almost all of the studies done are institution-based and do not expose the prevalence at the population level. This has contributed to policy gaps, leading to inadequate government investment on prevention and treatment of NCDs [7].

To understand the true burden of NCDs in Ethiopia, population based surveys are essential. Besides, it is time to assess the magnitude of NCDs and its associated factors at the population level for easy and cost-effective prevention.

3. Objective: The main objective of this study was to assess the magnitude and associated factors of NCDs at a DHSS site in the north-western part of the country.

For peer review only - http://bmjopen.bmj.com/site/about/guidelines.xhtml

Methods

4. **Study Design**

A population based cross sectional study was conducted from October to December 2014 at the Dabat HDSS site, Dabat District, northwest Ethiopia.

5. **Setting:** The district is located 820 km from Addis Ababa, the capital of Ethiopia. Based on the 2007 national census conducted by the Central Statistical Agency (CSA) [8], the district has a total projected population of 216,240 in 1,187.93 sq. km of land. Dabat has a population density of 122.49, which is greater than the zonal average of 63.76 persons per sq. km.

   The Dabat HDSS site was launched in the rural district of Dabat, by the Gondar College of Medical and Health Sciences (now University of Gondar) in 1995. In Ethiopia, there is inadequate vital events registration system to date; instead, population and housing census, national sample surveys, and health facility reports are the main sources of information on population health. Thus, Dabat HDS was established in order to produce a longitudinal data to aid in better health policy formulation, informed decision-making and health practice.

6. **Participants:** At the moment, 67,397 people live in the 16,053 households of the randomly selected 13 (9 rural and 4 urban) kebeles (the lowest administrative units) of the HDSS site. Household level data on the general socio-demography of family members, housing and environmental conditions, food security, chronic diseases, risk factors for non-communicable diseases, injury in the last two weeks, maternal and child health are updated every six months, that is, twice a year. All permanent residents in the DHS area were eligible to participate in the study. The majority of the residents live on subsistence agriculture, producing mainly food crops.

7. **Data sources/ measurement: A** structured, interviewer administered questionnaire was used to collect data on reported history of blood pressure, diabetes, cancer, heart disease, asthma, and history of other risk factors for chronic disease conditions (alcohol consumption, cigarette smoking, vegetable consumption, and physical activity). Data were collected by diploma graduate professionals who were experienced in data collection at the HDS center. The questionnaire was developed by considering questions

For peer review only - http://bmjopen.bmj.com/site/about/guidelines.xhtml

previously used at this site. The English version of the questionnaire was translated to Amharic and back translated to English by language experts to maintain the consistency of meaning.

8. **Sample size and sampling technique:** All permanent residents (67,397) of the HDSS site living in 16,053 households were included in the study. This study included all the permanent residents at Dabat HDS research center. The heads of households, mostly mothers, were interviewed to capture data on events (NCDs) that happened in the families. The outcome of this study, NCD, was ascertained using reported history of having at least one of the NCDs, such as diabetes mellitus, hypertension, cancer, asthma, and heart disease prior to data collection. Accordingly, a household member was labelled as having non-common chronic diseases if they had at least one reported NCD type. Socio-demographic characteristics (age, sex, ethnicity, religion, marital status, residence, wealth index and family size), food security status, and other health related factors were considered as independent variable.
9. **Quantitative variables:** Food security is defined as existing when "all people, at all times, have physical and economic access to sufficient, safe, and nutritious food to meet their dietary needs and food preferences for an active and healthy life". In order to assess the food security status of the households, an 18 item community food insecurity access scale assessment tool was adopted from the Household Food Insecurity Access Scale (HFIAS) Indicator Guide VERSION 3 and categorized into four levels, using the HFIS variables. A frequency of occurrence question was asked to determine whether the condition happened rarely (once or twice), sometimes (three to ten times), or often (more than ten times) in the past four weeks, to find out whether participants were food secure, mildly food insecure, moderately food insecure, or severely food insecure.

   Household wealth index was computed for urban and rural residents separately, using the Principal Component Analysis (PCA). Urban wealth status was calculated by considering properties like selected household assets, while only tropical livestock unit was used to determine rural income residents. In the PCA, an Eigen value of greater than one, Kaiser-Mayer-Olkin (KMO) distribution, and in the final model, common factor scores were summed and ranked into lowest, middle, second, fourth, and highest.

For peer review only - http://bmjopen.bmj.com/site/about/guidelines.xhtml

10. **Data quality control measures:** The quality of data was ensured by pre-testing the data collection tools on 5% of the participants in Debark district which has similar socio-demographic characteristics with Dabat district. Data was collected by 26 diploma graduate professionals and 5 individuals supervised the process. Supervision was also carried out at the spot by the research center leaders. Data collectors and supervisors had a long experience working for the Dabat DHS site. Training was given to both data collectors and supervisors on the questionnaire, content interviewing technique, purpose of the study, and on how to maintain confidentiality of information. The collected data were checked for completeness and clarity by assigned supervisors and the principal investigator.

11. **Data Analysis Procedures:** Data were entered into the HRS 2 software and exported to STATA version 14 for further analysis. The overall prevalence of NCDs was defined as the presence of any of the studied non-communicable chronic diseases during interview among the study population. The results were presented in the form of tables, figures, and summary statistics. Logistic regressions were used to assess the association of various factors and the outcome variable. The Bi-variable analysis with a crude odds ratio of 95% CI was used to see the crude effect of independent variables on NCDs. All independent variables that had a p-value of 0.20 in the Bi-variable analysis were fitted to the multivariable logistic regression analysis to control for possible confounding effects of variables. Variables which were found to be significant at a p-value 0.05 level and 95% CI were considered to be the determinant factors of NCDs.

12. **Ethical considerations**: Ethical clearance was obtained from the Institutional Review Board (IRB) of the University of Gondar before starting the actual data collection. Participants in the selected kebeles were recruited voluntarily after obtaining full information about the research and signed a written consent agreement. They were informed of their rights to withdraw from the study at any stage. Moreover, the confidentiality of information was guaranteed by using code numbers rather than personal identifiers and by keeping the data securely locked up. Individuals identified as cases of NCDs were referred to the nearby clinic for further treatment and follow-up.

Results

For peer review only - http://bmjopen.bmj.com/site/about/guidelines.xhtml

13. **Participants:** A total of 67,397 people were enrolled in this study. Women comprised about 50.77%. The majority (75.10%) of the study population was lived in areas. Among the study subjects, 42.96% (28,952) were under 15 years of age. Christianity is the dominant religion, accounting for 96.36%of the participants. The socio-demographic characteristics of the study population are depicted in Table 1.

14. **Descriptive data:** About 8.87% of the participants never drank alcohol in their life, while 88.2% and 2.88% were occasional and frequent consumers, respectively. About 99.5% of the study participants never smoked cigarettes in their lifetime, and 44.7% performed vigorous work related physical activities on daily bases (Fig1 and 2).

    Figure 1: Reported alcohol intake, smoking and physical activity by age group of Dabat HDS research center population, Northwest Ethiopia, 2016

    Figure 2: Frequency smoking, physical activity, and alcohol consumption of Dabat HDSS research center population, north-west Ethiopia, 2016

15. **Outcome data:** Participants who had NCDs were 1160 (1.7%) [95%CI: 1.62, 1.82], and according to the proportionate NCDs, morbidity ratio (morbidity fraction) was 404 (32.2%) heart disease, 401 (31.9%) hypertension, 347 (27.7%) asthma, 62 (4.9%) diabetes mellitus, and 40 (3.2%) cancer (Table 2). The mean (±SD) age of subjects with NCDs was 49.32 (±17.9). Of these, 746 (64.6%) were female with 48.96% in food insecure group. The prevalence of all types NCDs was slightly higher among women in all age groups. Out of the 16,035 households, 1146 (6.98%) had at least one NCD prevalence per household in the HDSS, and 40 (3.6%) of these had two NCDs cases per household.

16. **Factors:** After adjusting for a number of important covariates, the multivariable logistic regression analysis showed that increasing age, alcohol consumption, high income, living in urban areas, farming, work related physical activities, and severe food insecurity were associated with the odds of NCDs. Advanced age (≥ 65 year) (AOR= 19.6; 95% CI: 5.83 – 65.7), living in urban areas (AOR= 2.20; 95% CI: 1.83, 2.65), severe household food insecurity (AOR= 1.71; 95% CI: 1.37, 2.12), and High income (The odds NCD was 28% high among high income group compared with poor income) (AOR= 1.28; 95%CI: 1.02,

For peer review only - http://bmjopen.bmj.com/site/about/guidelines.xhtml

For peer review only

1.59),were significantly associated with reported history of NCDs, while low and moderate alcohol consumption (AOR= 0.36, 95% CI: 0.31, 0.42) and (AOR=0.33 95% CI: 0.22, 0.48), farming occupation (AOR= 0.72; 95% CI: 0.57, 0.91), and work related physical activities (AOR= 0.66; 95% CI: 0.50, 0.88) were inversely associated with NCDs (Table 3).

Discussion

Key result: The number of NCD cases seen at the Dabat HDS site was larger among the elderly; the proportions were considerably high among urban dwellers. A similar to another study (9) done in rural and urban Ethiopia, older age, physical inactivity, living in urban areas, farming, alcohol consumption, severe food insecurity, and low income were the strongest predictors of NCDs. [9]. Similar review study done in Sub-Saharan countries showed that NCDs are now the second most common causes of death after infectious diseases [10]. Our findings show that the occurrence of NCDs is significantly high among severely food insecure groups. This study is supported by a finding from Bangladesh which showed that persons with shorter stature are more likely to develop NCDs like diabetes and hypertension [11]. The WHO disease classification report shows that the disease phenotype encountered in Africa is very different from the West and most closely resembles malnutrition-related. Some researchers also indicated that early exposure to cereal products or solid foods might increase immune response, which could trigger the destruction of β-cells [12].

. Another finding shows that people who were small at birth and grew least during the first year of life had a chance of developing NCDs in later life [13]. This disease burden can affect the normal growth of the people, there-by developing early onset of non-communicable diseases in early-life and childhood. This exposure is likely to be responsible for the observed high rate of prevalence in this low economic status group [14]. Likewise, in our study a high level of NCDs were noted among individuals with high income.

This can be plausibly explained by the fact that people with better economic status will be more likely to favour mechanization which in turn leads to transition from physically active to less physically active sedentary lifestyle, keeping people indoors. Moreover, people with better

For peer review only - http://bmjopen.bmj.com/site/about/guidelines.xhtml

economic status will be more likely to follow unhealthy eating habits as they can afford diets richer in un-saturated fat, thereby being more susceptible to obesity and development of NCDs. [15, 16]. These data suggest that the burden of NCDs is increasing, perhaps as a result of demographic and epidemiological changes. Even though the country is undergoing a delayed transition, the burden of both malnutrition related and non-communicable diseases remains high [17, 18]. This double burden has a negative implication on major economic transitions [19].

The effect of ageing on NCD prevalence is already evident in Africa [20]. Aging causes a progressive decline in the strength of musculature, which causes muscle atrophy and the risk of developing CVD. In our finding, the prevalence of NCDs increases with advancing age. This finding is consistent with other reports from India, [15] as NCD epidemic matures, the onset will shift to younger age-groups. At aging, many more people are exposed to the risk factors for long periods until the complications develop and they experience the clinical syndromes of NCDs [17]. In Ethiopia, lack of awareness, distance to facilities, and un affordability of health services delay diagnoses for the elderly
[21].

Physical activity is more common in rural than urban regions of Africa because rural dwellers often rely on intense agricultural activities as their main occupation. In line with this, our finding shows that the prevalence of NCDs is low among farmers and physically active individuals which is consistent with the meta-analysis done on the risk factors for chronic non-communicable diseases in China, Ghana, Mexico, India, Russia, and South Africa[22]. Sedentary lifestyle and lack of physical activities accelerate the occurrence of NCDs; this condition is a growing challenge to sub-Saharan countries [23, 24]. Walking time and space are drastically reduced in an urban community as compared with a rural community. This reduction in physical activity associated with city life, partly explains the excessive prevalence of NCDs in urban areas. Studies show that lack of physical activity appears to be a significant risk factor for CVD in Sub-Saharan Africa [15, 17]. Reductions in physical activity and more sedentary modes of living parallel nutrition transition, producing an overall positive energy balance resulting in a paradigm of lifestyle transition [25].

For peer review only - http://bmjopen.bmj.com/site/about/guidelines.xhtml

In developing countries, rapid and unplanned urbanization and modernization resulting in socio-economic and behavioural changes in people directly affects the health of the urban population [17, 26]. The main difference in physical activity between the two types of community, however, is the use of walking in rural areas as means of transportation. A diet high in total fat, cholesterol, sugar and other refined carbohydrates, and low in polyunsaturated fatty acids and fibre, often accompanied by an increasingly sedentary life, characterize life in the urban community [26]. In other presumptions people exposed to periodic famines, which frequently occur in Africa, would through natural selection increase the frequency of certain genetic trait(s); these genes, also called "thrifty genes," will be expressed as individuals get exposed to undernutrition and starvation. This adaptation will enable individuals be able to cope with the shortage of energy by supressing body metabolism and limiting it only to sustaining the highly vital metabolisms of the body. However, when such individuals with activated thrifty genes from undernutrition experience improvement in their nutritional intake, the already supressed level of body metabolism may not be able to improve to metabolize the otherwise normal nutritional intake. This inability to sufficiently metabolize nutrients will lead to fat accumulation, dyslipidaemia, and diabetes despite the individual's relatively normal nutritional intake. Therefore, a continued discussion of proven cost-effective interventions of non-communicable chronic diseases, will be useful for improving public health policy [25].

Strengths of the study: This is the first community-based study examining the magnitude and associated factors of NCDs in the wider population, HDSS site, in Ethiopia.

- The findings of this study have provided important information that can be used to improve screening and care through medical evaluation to detect and manage NCDs.

17. **Limitations:** The limitation of this study is that it has used self-reported NCDs that may introduce recall and social desirability biases, and the morbidities reported might not be accurate diagnosis of the disease condition, because diseases might be miss-classified. Moreover, since this re-census is a general survey of the population, it may not provide in-depth information about specific diseases which have occurred in the community.

For peer review only - http://bmjopen.bmj.com/site/about/guidelines.xhtml

Another limitation of this study is that it used only a cross-sectional design which might not show temporal relationships; thus, the observed associations might not necessarily be causal. Therefore, a longitudinal research is needed to assess the relationships among the variables over time.

18. Conclusion: The number of NCD cases seen at the Dabat HDS site is higher among the elderly, and the proportions were considerably higher among the urban population. Effective and sustainable strategy for the prevention of chronic diseases throughout, implementing new intervention strategies by providing playgrounds to the urban community, improving the lively-hood of the rural community, and increasing access to healthy foods are highly essential.

Acknowledgement

We would like to acknowledge the University of Gondar and EPH CDC for the financial support. We extend our thanks to the Dabat District Health Office for their permission to conduct the study. We want to express our gratitude to all staff of the Dabat DHS and the study participants.

Authors' contributions

SM, GA, AG, YK, TA, MW, TA, MB, KA designed the study, SM, AS, MB, AT performed the analysis and interpretation of data and drafted the paper. SM, AS, MB, AT SM, GA, AG, YK, TA, MW, TA, MB, KA, revised the drafts of the paper. All authors prepared the draft manuscript, read and approved the final manuscript.

Funding: There are no funders to report for this submission

The authors declare no competing interests.

Data sharing statement: No additional data are available.

For peer review only - http://bmjopen.bmj.com/site/about/guidelines.xhtml

References

1. Habib SH, S.S., *Burden of non-communicable disease: Global overview. .* Diabetes Metab Syndr Clin Res Rev. , 2010. **4** (1): p. 41-47.
2. Miranda, J.J., et al., *Non-communicable diseases in low- and middle-income countries: context, determinants and health policy.* Trop Med Int Health, 2008. **13**(10): p. 1225-34.
3. Kankeu, H.T., et al., *The financial burden from non-communicable diseases in low- and middle-income countries: a literature review.* Health Res Policy Syst, 2013. **11**: p. 31.
4. Boutayeb, A., *The double burden of communicable and non-communicable diseases in developing countries.* Trans R Soc Trop Med Hyg, 2006. **100**(3): p. 191-9.
5. Lee, I.M., et al., *Effect of physical inactivity on major non-communicable diseases worldwide: an analysis of burden of disease and life expectancy.* Lancet, 2012. **380**(9838): p. 219-29.
6. Mulu, A., et al., *Epidemiological and clinical correlates of malaria-helminth co-infections in Southern Ethiopia.* Malar J, 2013. **12**: p. 227.
7. Murray, C.J. and A.D. Lopez, *Global mortality, disability, and the contribution of risk factors: Global Burden of Disease Study.* Lancet, 1997. **349**(9063): p. 1436-42.
8. *Summary and statistical report of the 2007 population and housing census, Ethiopia* in *(CSA).* 2007.
9. Tesfaye, F., *Epidemiology of Cardiovascular Disease Risk Factors in Ethiopia The rural-urban gradient* in *Department of Public Health and Clinical Medicine*. 2008, Umeå University: Umeå University, Umeå 2008:2004042.
10. Mbewu, A. and J.C. Mbanya, *Cardiovascular Disease*, in *Disease and Mortality in Sub-Saharan Africa*, D.T. Jamison, et al., Editors. 2006: Washington (DC).
11. Hoque, M.E., M.R. Khokan, and W. Bari, *"Impact of stature on non-communicable diseases: evidence based on Bangladesh Demographic and Health Survey, 2011 data"*. BMC Public Health, 2014. **14**: p. 1007.
12. Knip, M. and O. Simell, *Environmental triggers of type 1 diabetes.* Cold Spring Harb Perspect Med, 2012. **2**(7): p. a007690.
13. Martyn, C.N. and D.J. Barker, *Reduced fetal growth increases risk of cardiovascular disease.* Health Rep, 1994. **6**(1): p. 45-53.
14. Seligman, H.K., et al., *Food insecurity and glycemic control among low-income patients with type 2 diabetes.* Diabetes Care, 2012. **35**(2): p. 233-8.

For peer review only - http://bmjopen.bmj.com/site/about/guidelines.xhtml

15. Yoon, J., et al., *The Non-Communicable Disease Burden in Korea: Findings from the 2012 Korean Burden of Disease Study.* J Korean Med Sci, 2016. **31 Suppl 2**: p. S158-S167.

16. Mbanya, J.C., et al., *Diabetes in sub-Saharan Africa.* Lancet, 2010. **375**(9733): p. 2254-66.

17. Steyn, K. and A. Damasceno, *Lifestyle and Related Risk Factors for Chronic Diseases*, in *Disease and Mortality in Sub-Saharan Africa*, D.T. Jamison, et al., Editors. 2006: Washington (DC).

18. Adetunji, J. and E.R. Bos, *Levels and Trends in Mortality in Sub-Saharan Africa: An Overview*, in *Disease and Mortality in Sub-Saharan Africa*, D.T. Jamison, et al., Editors. 2006: Washington (DC).

19. Musaiger, A.O. and H.M. Al-Hazzaa, *Prevalence and risk factors associated with nutrition-related noncommunicable diseases in the Eastern Mediterranean region.* Int J Gen Med, 2012. **5**: p. 199-217.

20. Ayernor, P.K., *Diseases of ageing in Ghana.* Ghana Med J, 2012. **46**(2 Suppl): p. 18-22.

21. Sikder, S.S., et al., *Care-seeking patterns for fatal non-communicable diseases among women of reproductive age in rural northwest Bangladesh.* BMC Womens Health, 2012. **12**: p. 23.

22. Wu, F., et al., *Common risk factors for chronic non-communicable diseases among older adults in China, Ghana, Mexico, India, Russia and South Africa: the study on global AGEing and adult health (SAGE) wave 1.* BMC Public Health, 2015. **15**: p. 88.

23. Thornton, J.S., et al., *Physical Activity Prescription: A Critical Opportunity to Address a Modifiable Risk Factor for the Prevention and Management of Chronic Disease: A Position Statement by the Canadian Academy of Sport and Exercise Medicine.* Clin J Sport Med, 2016. **26**(4): p. 259-65.

24. Mbanya, J.C. and K. Ramiaya, *Diabetes Mellitus*, in *Disease and Mortality in Sub-Saharan Africa*, D.T. Jamison, et al., Editors. 2006: Washington (DC).

25. Zaman, M.M., et al., *Prevalence of risk factors for non-communicable diseases in Bangladesh: Results from STEPS survey 2010.* Indian J Public Health, 2016. **60**(1): p. 17-25.

26. C. Angkurawaranon, W.J., B. Chenthanakij, P. Doyle, D. Nitsch, *Urbanization and non-communicable disease in Southeast Asia: a review of current evidence.* Public health, October 2014. **128**(10): p. 886-895.

For peer review only - http://bmjopen.bmj.com/site/about/guidelines.xhtml

Table 1: Socio-demographic characteristics of the population of Dabat HDS research center, Northwest Ethiopia, 2016.

Variables		Frequency	Percent
Sex	Male	33,181	49.23
	Female	34,214	50.77
Age in years			
	Under 15	28,952	42.96
	15 to 24	13,289	19.72
	25 to 34	8,170	12.12
	35 to 44	6,322	9.38
	45 to54	4,720	7.00
	55 to 64	3,020	4.48
	65 and above	2,918	4.33
Residence			
	Rural	50,769	75.10
	Urban	16,835	16.84
Marital status			
	Under 10 years old	20,089	29.81
	Married	21,814	32.37
	Single	19,746	29.30
	Divorced	2,390	3.55
	Widowed	2,369	3.52
	Separated	917	1.36
	Cohabiting	68	0.10
Religion			
	Orthodox	64,940	96.36
	Muslim	2,444	3.63
	Others (Catholic, Protestant)	11	0.01
Ethnicity			
	Amhara	67,294	99.85
	Tigre	84	0.12
Others (Oromo,Agaw)		17	0.02
Education			
	Not on education (<7years)	13,672	20.29
	Unable to read and write	21,149	31.38
	Read and write	5,541	8.22
	Grade 1-4	10,960	16.26
	Grade 5-6	4,560	6.77
	Grade 7-8	3,590	5.33
	Grade 9-10	4,375	6.49
	Grade 11-12	1,957	2.90
	Grade 12 and above	1,591	2.36

For peer review only - http://bmjopen.bmj.com/site/about/guidelines.xhtml

Variables	Frequency	Percent
Occupation		
No occupation(under 10 years)	20,436	38.45
Students	13,955	26.26
Farmers	12,647	23.80
Employed permanent	1,951	3.67
Private job	1,167	2.20
Job seeker	1056	1.99
Merchant	656	1.23
House maid	623	1.17
Employed contract	328	0.62
Retired	296	0.56
Others(housewife, shepherd, disabled)	33	0.05
Location		
Low land	22,380	66.9
High land	45,224	33.1
Relation to the HH head		
HH head	16,082	23.86
Housewife	10,542	15.64
Son/daughter	34,702	51.49
Sister/brother	538	0.80
Mother/father	310	0.46
Grandson/granddaughter	3095	4.59
Other relative	1196	1.77
Other non-relative	930	1.38
Family size		
1-4	24,512	36.35
5-9	41,667	61.79
10-15	1,250	1.85
Wealth status		
Poorest quintile	9,475	14.58
Second quintile	11,344	17.45
Third quintile	13,031	20.05
Fourth quintile	14,593	22.45
Richest Quintile	16,577	25.47

For peer review only

For peer review only - http://bmjopen.bmj.com/site/about/guidelines.xhtml

Table 2: Proportion of Chronic Non-Communicable Diseases by age, and sex among Dabat HDS sit population, Northwest Ethiopia, 2016.

Variable	Gender	Reported cases age in Year n(%)						
		1-14	15-24	25-34	35-44	45-54	55-64	≥ 65
Diabetes	Female	1(0.01)	5 (0.08)	2 (0.05)	3(0.09)	3 (0.12)	7 (0.45)	5 (0.38)
mellitus	Male	4 (0.03)	7 (0.10)	7 (0.18)	5 (0.17)	4 (0.19)	4 (0.28)	5 (0.31)
Hypertension	Female	1 (0.010)	6(0.09)	22 (0.50)	30 (0.91)	49(1.90)	67 (4.26)	86 (6.56)
	Male	2 (0.01)	4 (0.06)	3 (0.08)	15 (0.50)	24 (1.12)	29 (2.0)	60 (3.73)
Heart Disease	Female	10 (0.07)	31(0.47)	54 (1.24)	50 (1.52)	66 (2.56)	43 (2.74)	47 (3.59)
	Male	4 (0.030)	12 (0.18)	14 (0.37)	14 (0.46)	19 (0.89)	12 (0.83)	27 (1.68)
Asthma	Female	2(0.01)	7(0.11)	32 (0.73)	41(1.24)	44(1.71)	36(2.29)	31(2.37)
	Male	4(0.03)	6(0.09)	21(0.55)	33(1.09)	32(1.50)	29 (2.0)	28(1.74)
Mental health	Female	12 (0.08)	26 (0.39)	27 (0.62)	18 (0.55)	19 (0.74)	11(0.70)	9(0.69)
	Male	11 (0.08)	21 (0.31)	16 (0.42)	14 (0.46)	16 (0.75)	3 (0.21)	5 (0.31)
Cancer	Female	2 (0.01)	0 (0.00)	2 (0.05)	6 (0.18)	10 (0.39)	0 (0.00)	6 (0.46)
	Male	1 (0.01)	2(0.03)	1(0.03)	1(0.03)	1 (0.05)	5 (0.35)	3 (0.19)

For peer review only - http://bmjopen.bmj.com/site/about/guidelines.xhtml

Table 3: Factors associated with NCDs of the population of Dabat HDSS research center, Northwest Ethiopia, 2016.

Variables	NCDs case n(%)	COR [95%CI]	AOR [95%CI]
**Sex**			
Female	746 (2.18)	1.00	1.00
Male	409 (1.23)	0.83 [0.74,0.93]	0.85 [0.69 - 1.03]
**Age in year**			
0 to 14	**30 (0.10)**	**1.00**	1.00
15 to 24	78 (0.59)	2.76 [2.20,3.45]	1.55 [0.47, 5.10]
25 to34	153 (1.87)	4.17 [3.32, 5.23]	3.64[1.09- 12.13] *
35 to44	188 (2.97)	4.73 [3.73,5.99]	6.33 [1.89- 21.2]**
45 to54	233 (4.94)	7.23 [5.74,9.12]	11.2 [3.36- 37.5] **
55 to64	209 (6.92)	10.1 [7.92,12.79]	16.1[4.79 - 53.8] **
≥ 65	264 (9.05)	26.27 [21.2,32.2]	19.6 [5.83 – 65.7] **
**Alcohol consumption**			
Never	340 (1.03)	1.00	1.00
Occasional	782 (2.35)	0.50 [0.42, 0.59]	0.36 [0.31 - 0.42] **
2 to 3 times a week	33 (3.04)	0.47 [0.31, 0.71]	0.33 [0.22 - 0.48] **
**Smoking status**			
No	1,110 (2.96)	1.00	1.00
Yes	6 (3.51)	1.73 [0.85, 3.52]	0.80 [0.34 - 1.91]
**Wealth status**			
Poor income	170 (1.80)	1.00	1.00
Low income	172 (1.52)	0.67 [0.57, 0.79]	0.90 [0.71 - 1.14]
Mid-level income	175 (1.34)	0.45 [0.38,0.55]	0.91 [0.72 - 1.16]
Better income	279 (1.91)	0.48 [0.41,0.57]	1.19 [0.96 – 1.49]
High income	336 (2.03)	0.35 [0.29,0.42]	1.28 [1.02 - 1.59] *
**Residence**			
Rural	538 (1.06)	1.00	1.00
Urban	617 (3.68)	1.06 [0.93,1.20]	2.20 [1.83 - 2.65] **
**Education status**			
Illiterate	10 (0.07)	1.00	1.00
Can read &write	646 (3.05)	12.549.25,16.99]	0.85 [0.20 - 3.52]
Primary school	140 (2.53)	6.274.41,8.91]	0.87 [0.21 – 3.61]
High school	55 (0.50)	2.48 [1.72, 3.56]	1.29 [0.31 – 5.39]
Diploma and above	304 (1.89)	3.15 [2.25, 4.40]	1.43 [0.35 – 5.89]

For peer review only - http://bmjopen.bmj.com/site/about/guidelines.xhtml

VARIABLES	NCDs case N (%)	COR [95%CI]	AOR [95%CI]
**Occupation**			
Under age	556 (1.60)	1.00	
Student	60 (0.43)	1.551.19,1.99]	0.84 [0.53 - 1.34]
Farmer	223 (1.76)	3.403.20,4.97]	0.72[0.57 – 0.91]*
Paid job	257 (5.44)	3.112.33,4.14]	1.13 [0.91 – 1.40]
Unemployed	14 (1.33)	2.92 [1.74,4.87]	0.66 [0.36 - 1.20]
Others	45 (13.72)	35.7 [25.38, 50.43]	1.34 [0.92 – 1.94]
**Doing Work related Physical activity**			
Under age	287 (0.84)	1.00	1.00
Sometimes	484 (2.96)	0.24 [0.21,0.28]	0.72 [0.59 - 0.86] **
Most of the time	384 (2.28)	0.17 [0.14,0.19]	0.64 [0.52 - 0.79] **
**Food security access**			
Food secure	565 (1.77)	1.00	1.00
Mildly food insecure	110 (1.51)	1.19 [0.96,1.46]	1.08 [0.86 - 1.34]
Moderately food insecure	296 (1.56)	1.71 [1.49, 1.96]	1.26 [1.07 - 1.48] *
Severely food insecure	136 (2.37)	2.70 [2.28,3.20]	1.71 [1.37 - 2.12] **
**Location of place**			
High land	924 (2.05)	1.00	1.00
Low Land	231 (1.03)	0.96 [0.85, 1.08	1.01 [0.84 - 1.22]
**Marital status**			
Under age	13 (0.06)	1.00	1.00
Marred	655 (3.00)	4.5 [4.29,6.93]	0.86 [0.20 - 3.61]
Single	82 (0.42)	4.07[3.17, 5.21]	0.37[0.09 – 1.57]
Divorced	142 (5.94)	16.1[12.2,21.3]	0.91 [0.21 - 3.88]
Widowed	234 (9.88)	23.9 [18.4, 31.2]	1.07 [0.25 – 4.55]
Separated	29 (2.94)	7.68 [4.94,11.96]	0.69 [0.15 – 3.06]
**Reported history of Injury**			
No	1139 (1.70)	1.00	1.00
Yes	16 (3.92)	1.90 [1.44, 3.82]	2.05 [0.15 – 3.34]

*:- P-value <0.05; **:- P-value <0.001

Other: - house wife and retired

For peer review only - http://bmjopen.bmj.com/site/about/guidelines.xhtml

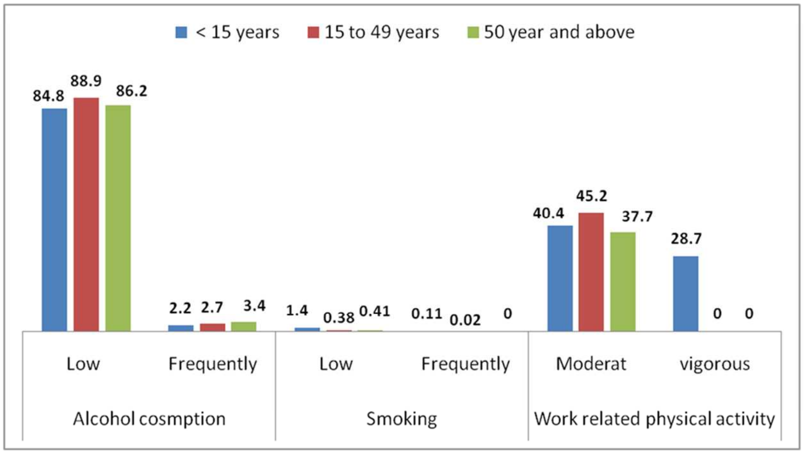

279x215mm (300 x 300 DPI)

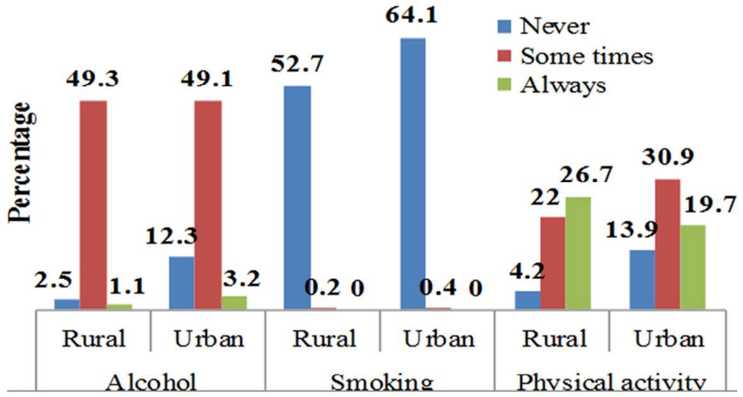

279x215mm (300 x 300 DPI)

For peer review only - http://bmjopen.bmj.com/site/about/guidelines.xhtml

Check list of items that is included in reports of our studies

	Item No.	Page No.
**Title**		**1**
**abstract**	**1**	**2**
**Introduction**		
Background/rationale	2	3
Objectives	3	4
**Methods**		
Study design	4	4
Setting	5	4
Participants	6	5
Data sources/ measurement	7	5
Study size	8	5
Quantitative variables	9	6
Data quality control measures	10	6
Data Analysis Procedures	11	6
Ethical statement	12	7
**Results**		
Participants	13	7
Descriptive data	14	7
Outcome data	15	8
Factors	16	8
**Discussion**		
Key results	17	8
Limitations	18	11
Conclusion	19	11

For peer review only
